# Estimates of aid for reproductive, maternal, newborn, and child health: findings from application of the Muskoka2 method, 2002–17

**DOI:** 10.1016/S2214-109X(20)30005-X

**Published:** 2020-02-05

**Authors:** Antonia Dingle, Marco Schäferhoff, Josephine Borghi, Miriam Lewis Sabin, Leonardo Arregoces, Melisa Martinez-Alvarez, Catherine Pitt

**Affiliations:** a Department of Global Health and Development, London School of Hygiene & Tropical Medicine, London, UK; b Medical Research Council Unit in The Gambia, London School of Hygiene & Tropical Medicine, London, UK; c Open Consultants, Berlin, Germany; d Partnership for Maternal, Newborn & Child Health, World Health Organization, Geneva, Switzerland

## Abstract

**Background:**

Four methods have previously been used to track aid for reproductive, maternal, newborn, and child health (RMNCH). At a meeting of donors and stakeholders in May, 2018, a single, agreed method was requested to produce accurate, predictable, transparent, and up-to-date estimates that could be used for analyses from both donor and recipient perspectives. Muskoka2 was developed to meet these needs. We describe Muskoka2 and present estimates of levels and trends in aid for RMNCH in 2002–17, with a focus on the latest estimates for 2017.

**Methods:**

Muskoka2 is an automated algorithm that generates disaggregated estimates of aid for reproductive health, maternal and newborn health, and child health at the global, donor, and recipient-country levels. We applied Muskoka2 to the Organisation for Economic Co-operation and Development's Creditor Reporting System (CRS) aid activities database to generate estimates of RMNCH disbursements in 2002–17. The percentage of disbursements that benefit RMNCH was determined using CRS purpose codes for all donors except Gavi, the Vaccine Alliance; the UN Population Fund; and UNICEF; for which fixed percentages of aid were considered to benefit RMNCH. We analysed funding by donor for the 20 largest donors, by recipient-country income group, and by recipient for the 16 countries with the greatest RMNCH need, defined as the countries with the worst levels in 2015 on each of seven health indicators.

**Findings:**

After 3 years of stagnation, reported aid for RMNCH reached $15·9 billion in 2017, the highest amount ever reported. Among donors reporting in both 2016 and 2017, aid increased by 10% ($1·4 billion) to $15·4 billion between 2016 and 2017. Child health received almost half of RMNCH disbursements in 2017 (46%, $7·4 billion), followed by reproductive health (34%, $5·4 billion), and maternal and newborn health (19%, $3·1 billion). The USA ($5·8 billion) and the UK ($1·6 billion) were the largest bilateral donors, disbursing 46% of all RMNCH funding in 2017 (including shares of their core contributions to multilaterals). The Global Fund and Gavi were the largest multilateral donors, disbursing $1·7 billion and $1·5 billion, respectively, for RMNCH from their core budgets. The proportion of aid for RMNCH received by low-income countries increased from 31% in 2002 to 52% in 2017. Nigeria received 7% ($1·1 billion) of all aid for RMNCH in 2017, followed by Ethiopia (6%, $876 million), Kenya (5%, $754 million), and Tanzania (5%, $751 million).

**Interpretation:**

Muskoka2 retains the speed, transparency, and donor buy-in of the G8's previous Muskoka approach and incorporates eight innovations to improve precision. Although aid for RMNCH increased in 2017, low-income and middle-income countries still experience substantial funding gaps and threats to future funding. Maternal and newborn health receives considerably less funding than reproductive health or child health, which is a persistent issue requiring urgent attention.

**Funding:**

Bill & Melinda Gates Foundation; Partnership for Maternal, Newborn & Child Health.

## Introduction

In the push towards universal health coverage, focused efforts are needed to ensure that reproductive, maternal, newborn, and child health (RMNCH) receives adequate funding. Although domestic and non-traditional health financing sources have rightly received increased attention in recent years,^1^, ^2^ aid remains important, particularly for low-income countries,^3^ and is a key pillar in achieving the ambitious targets of the Every Woman Every Child Global Strategy for Women's, Children's and Adolescents' Health (2016–2030).^4^ Estimates of aid for RMNCH can be used to hold donors and recipients accountable and to assess whether aid is sufficient, targeted to need, and effective.

Four methods to track aid for RMNCH have previously been used and compared:^5^ the G8 Muskoka method,^6^ the Countdown to 2015 approach,^7^ the Institute for Health Metrics and Evaluation (IHME) approach,^8^ and the Organisation for Economic Co-operation and Development (OECD) RMNCH policy marker.^9^ Although all of these approaches are designed to measure aid for RMNCH, their methods and estimates vary substantially. As previously described,^5^ any aid tracking approach comprises “trade-offs between simplicity, timeliness, precision, accuracy, efficiency, flexibility, replicability, and the incentives created”, and the most appropriate analytical choices depend on the objectives.

**Research in context**

**Evidence before this study**

In 2018, Pitt and colleagues conducted an in-depth comparison of the methods and findings of four existing approaches to track aid for reproductive, maternal, newborn, and child health (RMNCH). Important differences were found in the estimates and trends produced, particularly for individual donors and recipient countries. The Countdown to 2015 and G8 Muskoka (Muskoka1) approaches produced the largest and most similar estimates. The Countdown approach produced granular estimates, but it was time-consuming, lacked transparency, and has been discontinued. Muskoka1 has previously been widely used by donors and other stakeholders as an accountability tool, but its use has decreased in recent years and it was not designed for granular analyses. The Institute for Health Metrics and Evaluation produces annual reports of health sector aid, which include estimates of aid targeted towards RMNCH, exclusive of aid targeted towards HIV/AIDS, malaria, the humanitarian sector, and other areas. The Organisation for Economic Co-operation and Development introduced an RMNCH policy marker to its reporting system in 2014, which allows donors to indicate the proportion of the value of each disbursement that supports RMNCH; however, data are largely only available from 2013 onwards and many donors do not use the policy marker consistently, which restricts the usefulness of its findings. Several other initiatives exist to track narrower components of RMNCH and related health areas.

**Added value of this study**

We present Muskoka2, a new method for estimating the value of aid for RMNCH. Muskoka2 is a joint initiative of the Countdown to 2030 and the Partnership for Maternal, Newborn & Child Health and reflects an extensive stakeholder consultation process. It draws on the strengths of the previous Countdown to 2015 and Muskoka1 tracking approaches, which Muskoka2 is intended to supersede. We apply the Muskoka2 method to publicly available disbursement data to generate new estimates of aid for RMNCH from 2002 to 2017. Our analysis provides an important basis for holding donors accountable for their commitments and for assessing whether aid is sufficient, equitable, and effective.

**Implications of all the available evidence**

The Muskoka2 method for tracking aid for RMNCH retains the advantages of Muskoka1, and incorporates eight innovations to improve precision. Our findings indicate an increase in aid for RMNCH in 2017 relative to all previous years, increased prioritisation of low-income countries, and many new donors publicly reporting their aid; however, the ongoing neglect of maternal and newborn health requires urgent attention.

In May, 2018, the Partnership for Maternal, Newborn & Child Health and the Countdown to 2030 convened donors and other stakeholders, who requested a single, agreed method that produces timely, accurate, predictable, and transparent estimates to permit analyses from both donor and recipient-country perspectives.^10^ The donors and stakeholders also requested that the new method should allow donors to track their own progress against commitments and should help recipient countries and stakeholders to understand and assess aid.^10^ A Technical Working Group for Tracking Financing for Sexual, Reproductive, Maternal, Newborn, Child and Adolescent Health was established to improve aid tracking for RMNCH by building on the strengths of existing approaches^5^ in ways that would meet stakeholders' needs (appendix p 2). From this Working Group emerged the new Muskoka2 method, which estimates the monetary value of aid for the reproductive and sexual health of non-pregnant women, and the health of pregnant and post-partum women and of children younger than 5 years.

We aim to describe the consultation process through which Muskoka2 was developed, explain the new method and how it innovates beyond the original Muskoka approach (henceforth referred to as Muskoka1), and present estimates of levels and trends in aid for RMNCH from 2002 to 2017, with a focus on the latest estimates for 2017.

## Methods

### Development of Muskoka2

Muskoka1 was developed by the G8 Health Working Group in advance of the G8 Summit in Muskoka, Canada, in 2010. It was widely used by G8 countries^6^, ^11^ and other stakeholders—including in annual Partnership for Maternal, Newborn & Child Health reports^12^—to track whether donors had fulfilled their commitments to RMNCH; however, its use has decreased in recent years and it was not designed to produce global or recipient-specific estimates.^5^, ^13^

The Technical Working Group for Tracking Financing for Sexual, Reproductive, Maternal, Newborn, Child and Adolescent Health developed Muskoka2 through an iterative consultation process with representatives of bilateral donors (ie, governments that provide aid to recipient countries); multilateral institutions (ie, international organisations, the members of which include multiple governments), including WHO and the OECD; and academia and civil society. Three meetings were held in 2018, during which participants reviewed and finalised the choice of assumptions, data sources, and approaches underpinning Muskoka2.

The Muskoka2 method was designed to retain the speed, simplicity, transparency, and stakeholder buy-in of Muskoka1, while incorporating eight innovations to improve accuracy and permit more granular, recipient-specific analyses, which largely draw on the previous Countdown approach (table; appendix pp 2–6).^7^ The Muskoka2 method was presented at the formal meeting of the OECD Development Assistance Committee in November, 2018, and received support.^13^

**Table:** Comparison of Muskoka1 and Muskoka2

	**Muskoka1**	**Muskoka2**
Recipient-specific and year-specific imputed percentages	Not used; the same imputed percentages are applied to aid in all years and for all recipient countries	Percentage of funding for HIV/AIDS, malaria, tuberculosis, and general budget support that is counted towards RMNCH estimates varies by year and recipient country
Disaggregation of estimates by beneficiary group	Only aid for RMNCH estimated	Aid for RMNCH disaggregated into aid for reproductive health, maternal and newborn health, and child health, which permits estimates of aid for reproductive health per woman of reproductive age, aid for maternal and newborn health per birth, and aid for child health per child
Regional and unspecified recipients	Not included in aid estimates for recipient countries	Recipient countries are assumed to receive funding for regional and unspecified recipients in proportion to their receipt of country-specific funding
Humanitarian funding	Excluded	Relevant share included
Private flows	Excluded	Included if reported
Treatment of disbursements from multilateral institutions	Not originally included; later, purpose-code-based percentages were applied to disbursements from all multilateral institutions	Revised institution-specific percentages applied to all funding from three institutions with RMNCH-specific mandates (Gavi, the Vaccine Alliance, UN Population Fund, and UNICEF) and purpose-code-based percentages applied to disbursements from all other institutions
Crediting bilateral donors for core contributions to multilateral institutions	Fixed percentage of core contributions to ten multilaterals included in estimates of aid from each bilateral donor	Percentage of core contributions to all multilaterals included in estimates of aid from each bilateral donor, on the basis of each multilateral's annual disbursements
Communication of uncertainty	None	Simple, wide bounds, and disaggregated reporting

RMNCH=reproductive, maternal, newborn, and child health.

### Muskoka2 method and data sources

Muskoka2 estimates the monetary value of funding that directly influences RMNCH outcomes, rather than only funding earmarked for RMNCH. Muskoka2 therefore includes shares of funding targeted at specific diseases, such as HIV^14^, ^15^ and malaria,^15^, ^16^ and at health system strengthening^17^ and the water and sanitation sector.^18^, ^19^ In addition, shares of funding for the humanitarian sector are included because humanitarian activities encompass health and water and sanitation interventions, including some directed specifically at RMNCH.^5^ Exclusion of humanitarian aid has also been shown to bias aid estimates for crisis-affected recipient countries.^5^ Muskoka2 consists of a transparent, automated algorithm applied to aid data reported to the OECD's Creditor Reporting System (CRS) aid activities database, which can generate estimates globally, by year, by recipient country, and by donor. The CRS avoids double-counting aid flows from bilateral donors to and through multilateral institutions by considering multilaterals as the donor of disbursements from their core budgets and bilaterals as the donor of disbursements over which they retain control of the purpose and recipient country.^20^

**Figure 1:**
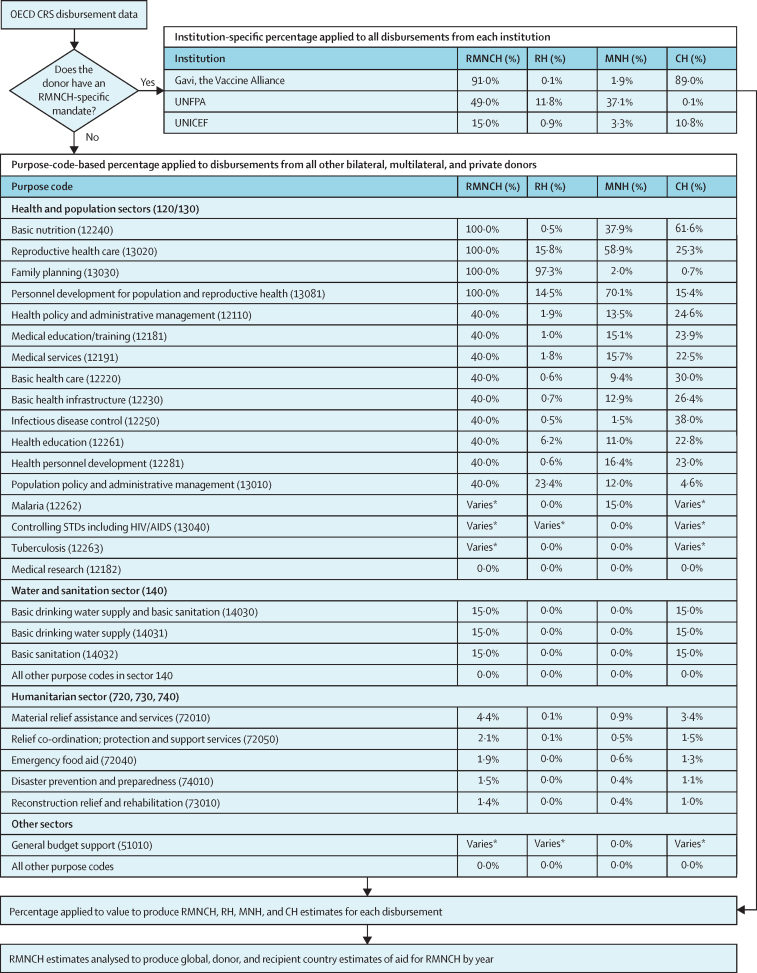
Flow diagram of the Muskoka2 method CH=child health. CRS=Creditor Reporting System. MNH=maternal and newborn health. OECD=Organisation for Economic Co-operation and Development. RH=reproductive health. RMNCH=reproductive, maternal, newborn, and child health. STD=sexually transmitted disease. UNFPA=UN Population Fund. *The proportion of funding in the malaria, HIV, tuberculosis, and general budget support purpose codes that is considered to support RMNCH varies by recipient country and year on the basis of publicly available data on either disease burden or government expenditure on health.

Muskoka2 uses six of 87 variables in the CRS: donor, year, disbursement amount, flow type, recipient, and purpose code. The purpose code identifies the sector and more specific objective of the funded activities, but it cannot provide a clear picture of RMNCH funding alone (figure 1).^21^ The flow type variable was used to define aid to include official development assistance (ODA) grants and loans and private development finance, and to exclude equity investments and other official flows. An aid recipient can be one of 138 specific recipient countries, one of 16 geographical regions, or unspecified (which often refers to institutional support to international non-governmental organisations or initiatives). We analyse disbursements, which reflect the “actual international transfer of financial resources, or of goods or services”.^22^

The number of donors reporting any disbursement data increased from 33 in 2002 to 114 in 2017, indicating a substantial number of missing donor-years of disbursement data (appendix pp 7–12). However, for the aid activities reported and relevant to the analysis, the donor, year, recipient, flow type, and purpose code fields do not contain missing data and blank disbursement values are assumed to reflect a true absence of disbursement (eg, commitment reporting).

Additional data sources were used in Muskoka2 to inform the proportion of aid to count towards RMNCH estimates. These sources included data on disease burden,^23^, ^24^ demography,^23^, ^24^ and government health expenditure,^25^ as well as the Countdown ODA+ dataset (2003–13).^7^ The latter was developed through largely manual coding of an earlier (January, 2017) version of the CRS according to an RMNCH activity framework. This coding used the same data as Muskoka2, as well as additional information in the project title and short and long description fields in the CRS, to generate estimates of aid for reproductive health, maternal and newborn health, and child health.^7^ To understand individual donors' contributions to RMNCH for Muskoka2, the OECD data table, Members' total use of the multilateral system (accessible from the CRS database webpage), was also used.

### Generating Muskoka2 estimates

Like Muksoka1, Muskoka2 generates estimates of aid for RMNCH by counting a percentage (0–100%) of the value of each disbursement in the CRS that benefits RMNCH (figure 1). With the exception of three donors (detailed later), the percentage applied is determined by the CRS purpose code (figure 1). Aid categorised in 25 of the 223 purpose codes is considered to benefit RMNCH and assigned a non-zero percentage. The percentages counted towards RMNCH are the same as for Muskoka1 for 16 of these 25 purpose codes.

For four of the 25 purpose codes—malaria, HIV/AIDS, tuberculosis, and general budget support—the percentages follow the same logic as those used in Muskoka1, but they are allowed to vary based on data on the disease burden,^23^, ^24^ demography,^23^, ^24^ and government health expenditure^25^ in each recipient country and year (figure 1). This method is consistent with the Countdown approach and accounts for the large variations in how much funding for each of these four areas benefits RMNCH across recipient countries and years. For example, in some countries, the malaria burden is borne almost exclusively by children and pregnant women, with the majority of malaria funding directly benefiting RMNCH. Given the substantial contribution of HIV and malaria funding to RMNCH, there is particular value in gaining additional precision in estimates.

Muskoka2 also includes a percentage of aid in all five humanitarian sector purpose codes, none of which had been included in Muskoka1. Percentages were based on the proportion of disbursements in that purpose code that benefited RMNCH in the Countdown dataset (2003–13).^7^ For example, 4·4% of aid for material relief assistance and services (purpose code 72010) over the period 2003–13 was counted towards RMNCH, and Muskoka2 applies this same percentage to disbursements in this purpose code (figure 1).

For Gavi, the Vaccine Alliance, the UN Population Fund (UNFPA), and UNICEF, the Working Group suggested the percentages based on purpose codes would underestimate their contributions to RMNCH. Fixed percentages of aid from Gavi (91%), UNFPA (49%), and UNICEF (15%) were therefore considered to benefit RMNCH (figure 1), reflecting the share of their disbursements benefiting RMNCH in the Countdown dataset.

In addition to generating estimates of aid for RMNCH, Muskoka2 also provides a breakdown of aid for reproductive health (defined as the reproductive and sexual health of non-pregnant women), maternal and newborn health (defined as the health of pregnant and post-partum women and babies younger than 1 month), and child health (defined as the health of children aged 1 month to 5 years; figure 1). For malaria, HIV, tuberculosis, and general budget support funding, our approach to generating RMNCH percentages already reflected these separate population groups because they were based on demographic and age-specific disease burden data. To disaggregate other RMNCH funding, we analysed the Countdown dataset^7^ to determine the proportion of aid for RMNCH that the Countdown approach estimated would benefit reproductive health, maternal and newborn health, and child health within each purpose code and for each of the three institutions with RMNCH-specific mandates.

To obtain a complete picture of aid from bilateral (government) donors, both the aid they provide to recipient countries and the core contributions they make to multilateral organisations must be considered. Muskoka2 uses estimates of core contributions to each multilateral from the OECD data table, Members' total use of the multilateral system. Muskoka2 calculates the proportion of core contributions to each multilateral that benefits RMNCH as the proportion of all disbursements from the relevant multilateral that is estimated to benefit RMNCH each year. For example, because 40% of the value of disbursements from the Global Fund in 2017 were considered to support RMNCH, 40% of each bilateral donor's core contributions to the Global Fund in 2017 were counted towards that bilateral donor's RMNCH contribution. We only apply this approach in comparing individual donors' RMNCH disbursements; estimates of global aid and aid for individual recipient countries are based exclusively on the CRS and therefore avoid double-counting of multilateral aid.

To generate and compare estimates of aid for RMNCH to individual recipient countries, Muskoka2 includes a share of funding for regional and unspecified recipients. Muskoka2 uses the same approach as the Countdown to 2015; countries are assumed to receive regional and unspecified funds in the same proportion as country-specific funding. For example, Nigeria received 11% of all country-specific RMNCH funding for sub-Saharan African countries in 2017, so Nigeria is also assumed to benefit from 11% of regional RMNCH funding for sub-Saharan Africa in 2017.

### Estimates of aid for RMNCH

The Muskoka2 method was used to produce global estimates of aid for RMNCH and disaggregated annual estimates of aid for reproductive health, maternal and newborn health, and child health at the global, donor, and recipient-country levels, as well as by purpose code and country income group for the years 2002–17.^26^ We applied Muskoka2 to disbursement data for 2002–17 in the December, 2018, version of the OECD CRS, which reflects reporting from 55 bilateral, 65 multilateral, and 28 private donors. We also analysed funding by donor for reproductive health, maternal and newborn health, and child health for the 20 largest donors. We examined aid for RMNCH by recipient for the 16 countries with the greatest health needs, defined as the six countries with the worst levels in 2015 on each of seven indicators: maternal mortality rate, number of maternal deaths, neonatal mortality rate, number of neonatal deaths, mortality rate of children younger than 5 years, number of deaths of children younger than 5 years, and female life expectancy.^27^ This definition of RMNCH need results in 16 rather than 42 countries because many of these 16 countries have some of the worst levels across multiple indicators. Estimates of aid per recipient are presented in aggregate and per relevant population: women of reproductive age, births, and children younger than 5 years.^24^

We describe aid estimates for 2017 and compare with estimates for 2016, 2012, and 2002. We restrict each comparison to aid from donors that reported a non-zero disbursement of any flow type in any sector for both years compared. This restriction avoids the reporting bias that could be introduced in comparing funding levels in years in which different donors reported their disbursements. For completeness, we also show all reported data.

Uncertainty is inherent in estimates of aid for RMNCH. We identified potential lower and extreme upper bound estimates to reflect uncertainty in the proportion of aid in each purpose code that benefits RMNCH. At the lower bound, we include 100% of aid in purpose codes that entirely support RMNCH, namely reproductive health, family planning, basic nutrition, and personnel development for population and reproductive health. At the extreme upper bound, all aid for the health, humanitarian aid, and water and sanitation sectors is included. We reflect uncertainty in methods for crediting donors for their core contributions to multilaterals by presenting these estimates separately from donors' direct disbursements to recipients. Similarly, in estimates for individual recipient countries, we distinguish between funding disbursed directly to each country, and regional and unspecified funding that we assume to benefit each country. To understand the impact on estimates of the changes introduced in Muskoka2, we applied the Muskoka1 methods^6^ to the same data and compared the findings with our Muskoka2 estimates.

Data were summarised using Microsoft SQL Server Management Studio 2014 and analysed in Microsoft Excel 2019. Estimates are presented in constant 2016 US$ using the Development Assistance Committee deflators, which account for variation in inflation in donors' currencies and exchange rates.

### Role of the funding source

The funder of the study had no role in study design, data collection, data analysis, data interpretation, or writing of the report. The corresponding author had full access to all of the data and the final responsibility for the decision to submit for publication.

## Results

**Figure 2:**
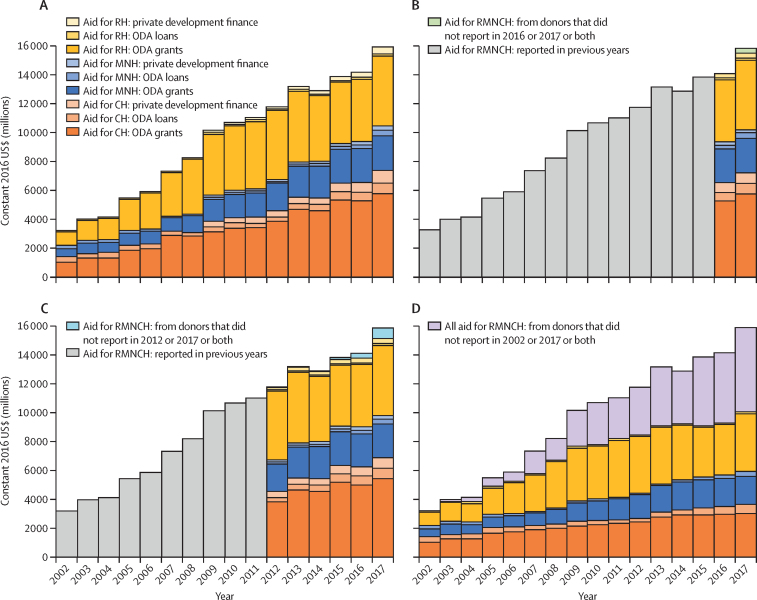
Trends in aid for RMNCH by beneficiary group and type of aid flow, 2002–17 Trends are presented for all reported data (A), and for data from donors reporting in 2016 and 2017 (B), 2012 and 2017 (C), and 2002 and 2017 (D). CH=child health. MNH=maternal and newborn health. ODA=official development assistance. RH=reproductive health. RMNCH=reproductive, maternal, newborn, and child health.

After 3 years of stagnation, reported aid for RMNCH reached $15·9 billion (constant 2016 USD, figure 2A) in 2017, the highest amount ever reported, and comprised 8% of all-sector aid (appendix p 13). Among donors that reported for both 2016 and 2017, aid for RMNCH increased by $1·4 billion (10%) to $15·4 billion between 2016 and 2017 (figure 2B). At the lower bound, our estimate of aid in the four RMNCH-specific purpose codes increased by 9% over the same period to $4·1 billion (appendix p 14). At the upper bound, health and population sector aid increased by 12% to $26·5 billion, water and sanitation sector aid decreased by 5% to $6·7 billion, and humanitarian sector aid increased by 14% to $28·2 billion (appendix p 14). All-sector aid increased by 4% to $189·9 billion; therefore, RMNCH comprised an increasing share of all-sector aid (appendix p 13).

Among donors that reported for both 2002 and 2017, aid for RMNCH tripled from 2002 to 2017 (figure 2D), as did aid to the health and population sector and the water and sanitation sector, whereas aid to the humanitarian sector increased by 4·5 times and all-sector aid doubled (appendix p 13). From 2002 to 2009, RMNCH funding from these donors increased at an average annual rate of 13%, compared with 3% from 2009 to 2016, before increasing by 8% in 2017 (figure 2D).

In 2017, child health received nearly half of all aid for RMNCH (46%, $7·4 billion), followed by reproductive health (34%, $5·4 billion), and maternal and newborn health (19%, $3·1 billion). Among donors reporting for both 2002 and 2017, aid for child health decreased from 44% of all aid for RMNCH ($1·4 billion) in 2002 to 37% ($3·7 billion) in 2017; aid for reproductive health increased from 32% ($1·0 billion) to 41% ($4·1 billion); and maternal and newborn health decreased from 24% ($762 million) to 22% ($2·3 billion) in 2017 (appendix p 15).

In 2017, aid directed towards HIV/AIDS comprised the largest share of aid for RMNCH (24% of total; $3·8 billion), followed by basic health care (12%; $1·9 billion), reproductive health care (12%; $1·8 billion), and infectious diseases (10%; $1·6 billion). Among donors that reported for both 2016 and 2017, the $1·5 billion increase in aid for RMNCH primarily reflected increases in aid directed towards HIV/AIDS (24% of the increase) and basic health care (21%; appendix p 17). In 2017, aid for reproductive health was comprised primarily of aid directed towards HIV/AIDS (69%) and family planning (19%); aid for maternal and newborn health was comprised largely of aid categorised in the reproductive health care purpose code (35%), basic nutrition (14%), malaria (11%), and health policy and administrative management (9%); and aid for child health was categorised as basic health care (23%), infectious diseases (20%), the humanitarian sector (10%), malaria (10%), and basic nutrition (9%; appendix p 18).

In 2017, 82% of aid for RMNCH was provided as ODA grants, 8% as ODA loans, and 10% as private development finance. From 2012 to 2017, the share of aid for RMNCH provided as ODA grants by donors reporting in both years decreased from 89% to 83%; private flows increased from 6% to 8%; and ODA loans increased from 4% to 8%. No private donors reported their funding prior to 2009 and many private donors only reported their aid for 2017 (appendix p 12).

**Figure 3:**
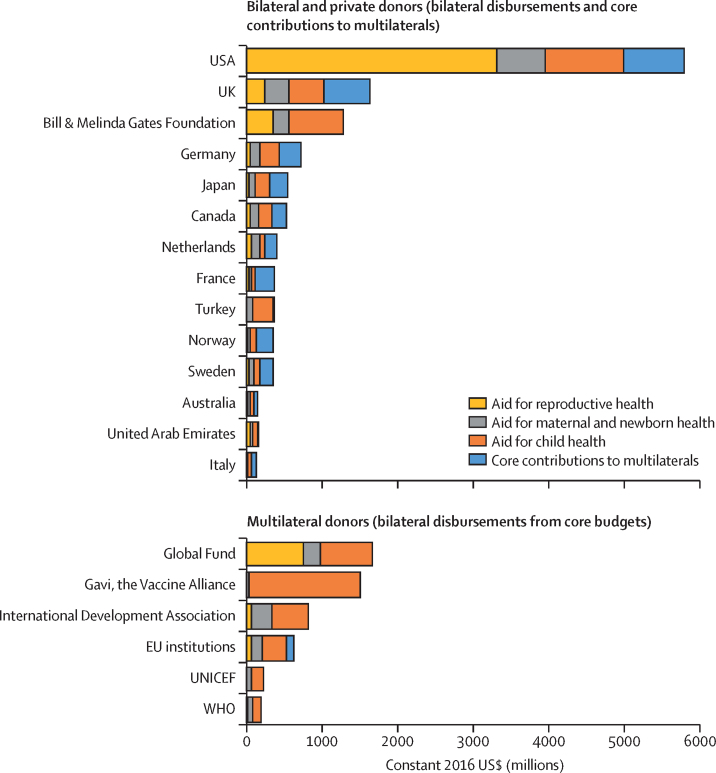
20 largest donors of aid for reproductive, maternal, newborn, and child health in 2017 Bilateral donors' core contributions to multilaterals overlap with the funds shown as disbursements from multilaterals for reproductive health, maternal and newborn health, and child health.

The largest bilateral and private donors in 2017 (including core contributions to multilaterals) were the USA ($5·8 billion, 36% of all aid for RMNCH), the UK ($1·6 billion, 10%), and the Bill & Melinda Gates Foundation ($1·3 billion, 8%; figure 3). Two emerging donors were among the 20 largest donors: Turkey disbursed $359 million (2%) for RMNCH, 99% of which provided humanitarian aid to Syria, and the United Arab Emirates disbursed $142 million (1%), half of which ($72m) reflected shares of general budget support.

Bilateral donors supported the multilateral system to differing degrees. Whereas the USA provided 14% ($806 million) of its aid for RMNCH in 2017 as core contributions to multilaterals, the UK provided 37% ($601 million) and France provided 70% ($261 million) in this manner. Multilaterals disbursed substantial sums from their core budgets, making them important donors in their own right; most notable were the Global Fund ($1·7 billion, 10% of all aid for RMNCH) and Gavi ($1·5 billion, 9%; figure 3).

To examine donor disbursements by beneficiary group, we excluded bilateral donors' core contributions. For reproductive health, the same few donors (with the exception of Gavi) provided the most aid, but the USA's role—largely driven by its HIV funding—was more dominant, accounting for 61% ($3·3 billion) of all reproductive health disbursements in 2017. For maternal and newborn health, the USA was also the largest donor, but it provided a smaller share of aid than for reproductive health (21%, $639 million), and was followed more closely by the UK (10%, $321 million) and other donors. For child health, Gavi was the largest donor (20%, $1·5 billion), followed by the USA (14%, $1·0 billion), the Gates Foundation (10%, $714 million), and the Global Fund (9%, $684 million; figure 3)

**Figure 4:**
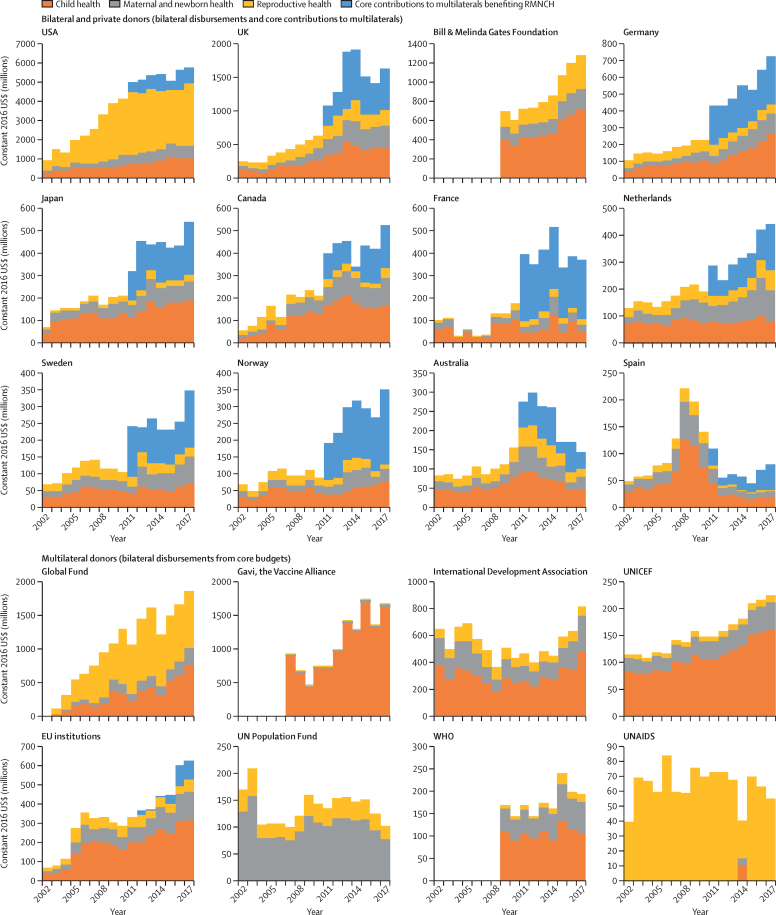
Trends in aid for RMNCH for each of the 20 largest donors, 2002–17 Donors were ranked on the basis of cumulative RMNCH disbursements over the period. Data on core contributions to multilateral institutions are only available from 2011 onwards. Bilateral donors' core contributions to multilaterals overlap with the funds shown as disbursements from multilaterals for reproductive health, maternal and newborn health, and child health. Gavi, the Gates Foundation, and WHO did not report data for all years. RMNCH=reproductive, maternal, newborn, and child health.

Aid from the USA drove the rapid increase in aid for RMNCH from 2002 to 2009 and the relative stagnation until 2016 (figure 4). The substantial increase in aid from 2016 to 2017 reflected a collective effort, in which the top ten RMNCH donors over the 2002–17 period all increased their aid. However, aid from France, Australia, UNFPA, WHO, and UNAIDS decreased in 2017, and aid from the UK and Gavi peaked in 2014 and 2015, respectively. WHO and the Gates Foundation did not report to the CRS on disbursements before 2009, and Gavi did not report before 2007.

**Figure 5:**
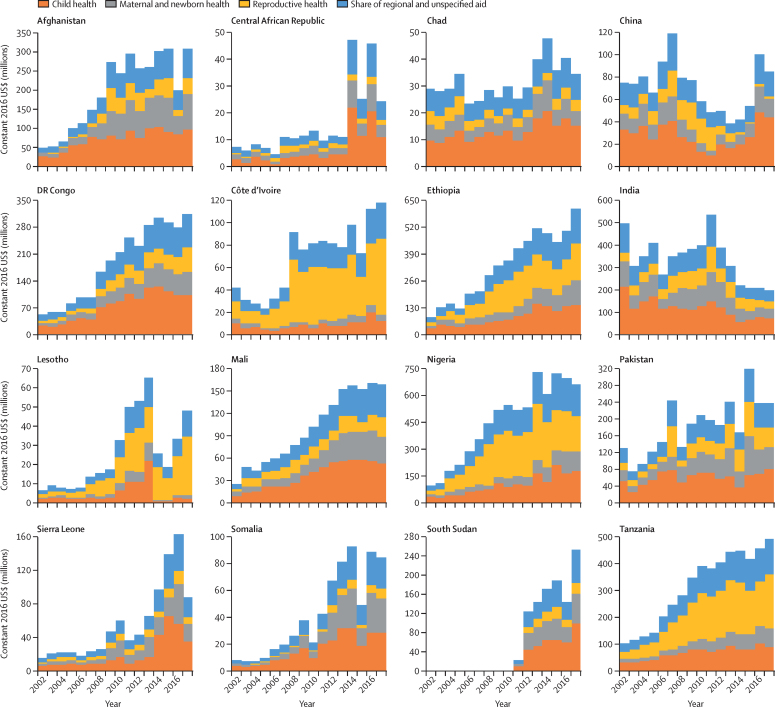
Trends in aid for RMNCH for each of the 16 recipient countries with greatest RMNCH need, 2002–17 Countries are ordered alphabetically. Data are restricted to donors that reported disbursements in both 2002 and 2017. RMNCH=reproductive, maternal, newborn, and child health.

Nigeria received the most aid for RMNCH in 2017, with 7% ($1·1 billion) of the total aid for RMNCH, followed by Ethiopia (6%, $876 million), Kenya (5%, $754 million), Tanzania (5%, $751 million), and Democratic Republic of the Congo (4%, $623 million; appendix p 21). The 16 highest-need countries collectively received 38% of aid for RMNCH in 2002 and 37% in 2017 from donors reporting in both years. For most of these recipients, RMNCH funding increased substantially from 2002 to 2017 (figure 5). However, aid received by India decreased from $497 million in 2002 to $268 million in 2006, before peaking in 2011 at $536 million and decreasing again to $203 million in 2017. Aid for RMNCH received by China peaked in 2007 at $119 million, decreased to a low of $39 million in 2013, and increased again to $86 million by 2017. Several of the 16 highest-need countries, notably the Central African Republic, Chad, Lesotho, and Pakistan, experienced substantial volatility in aid for RMNCH over time. For example, Pakistan's aid for RMNCH increased by 69% from 2006 to 2007, decreased by 46% in 2008, increased by 43% in 2009, and later increased by 89% from 2014 to 2015 before decreasing by 25% in 2016.

**Figure 6:**
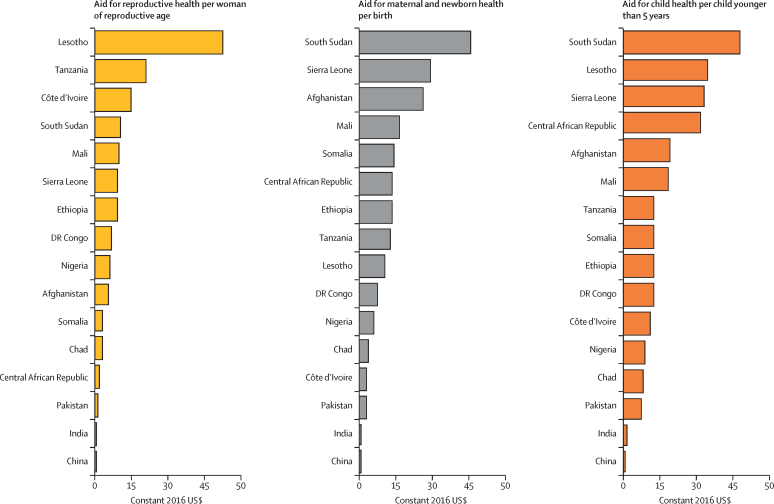
Aid for reproductive health, maternal and newborn health, and child health by health need for 16 recipient countries with the greatest RMNCH need, 2017 RMNCH need is defined here as the six countries with the worst levels in 2015 on each of seven health metrics. Because many of the same countries have the worst indicators across the seven metrics, this definition results in a list of 16 rather than 42 countries. RMNCH=reproductive, maternal, newborn, and child health.

The share of aid for RMNCH received by low-income countries increased steadily from 31% in 2002 to 52% in 2017. Low-income countries have received a larger proportion of the aid than lower-middle-income countries since 2012 (appendix p 16). Regional and unspecified recipients received 28% of aid for RMNCH in 2002 ($897 million) and 27% in 2017 ($4·3 billion; appendix p 17). Excluding this regional and unspecified funding, aid for reproductive health per woman of reproductive age varied across the 16 highest-need countries from $53 per woman in Lesotho to $0·01 per woman in China; aid for maternal and newborn health varied from $31 per birth in South Sudan to $0·20 per birth in China; and aid for child health varied from $64 per child younger than 5 years in South Sudan to $0·60 per child in China (figure 6).

Muskoka2 produced larger global estimates of aid for RMNCH than Muskoka1 for every year from 2003 to 2017 (appendix p 22). For 2017, the Muskoka2 global estimate ($15·9 billion) was $2·7 billion (20%) higher than that of Muskoka1 ($13·2 billion). The inclusion of relevant shares of aid from private donors accounted for $1·6 billion (10%) and from the humanitarian sector accounted for $976 million (6%) of the Muskoka2 estimate for 2017; neither of these aid flows were included in Muskoka1 estimates (table). For 2017, Muskoka2 included 5% ($173 million) more HIV/AIDS funding, 46% ($828 million) less malaria funding, 70% ($135 million) less tuberculosis funding, and 34% ($79 million) less general budget support in RMNCH estimates than Muskoka1. The changes to the treatment of multilaterals with RMNCH-specific mandates led Muskoka2 to include 147% more GAVI funding ($893 million), 17% more UNFPA funding ($15 million), and 45% more UNICEF funding ($70 million) in RMNCH disbursement estimates than Muskoka1. Muskoka2 estimates of aid for RMNCH (2002–17) were 4% ($2 billion) higher for the USA, similar for the UK, and 22% ($4 billion) lower for the Global Fund than Muskoka1. Over 2002–17, Muskoka2 estimates were 15% ($958 million) higher for Nigeria, 11% ($589 million) higher for Ethiopia, and 9% ($425 million) higher for Kenya than Muskoka1 estimates (appendix pp 23–25).

## Discussion

Muskoka2 is a new method for tracking aid for RMNCH that retains the speed, simplicity, transparency, and stakeholder buy-in of the original Muskoka method, and incorporates eight innovations to improve precision.^10^ Muskoka2 produces disaggregated estimates for reproductive health, maternal and newborn health, and child health and allows analysis at the individual donor and recipient-country level. The approach remains accessible for non-technical audiences and can quickly generate estimates through a Microsoft Excel template, which is publicly available and is being piloted with other users to develop user-friendly guidance. Muskoka2 harmonises efforts by the Countdown to 2030 and Partnership for Maternal, Newborn & Child Health to track aid for RMNCH.^10^, ^13^

Our estimate of a 10% increase in aid for RMNCH from 2016 to 2017 is encouraging, but the $1·4 billion increase is small relative to the $33·3 billion annual funding gap for achievement of the women's, children's, and adolescents' health targets in the Every Woman Every Child Global Strategy^4^ and health-related Sustainable Development Goals.^28^ Low-income countries, including those in Africa, received an increasing share of aid for RMNCH from 2002 to 2017, whereas aid stagnated for India and China, where large numbers of women and children do not have effective access to health services. Aid for maternal and newborn health increased in absolute terms, but accounted for only 19% of all aid for RMNCH in 2017, which is less than half of that for child health. This disparity requires urgent attention because progress in reducing maternal and neonatal mortality has lagged behind progress in child mortality, and the number of newborn deaths is now nearly equal to the number of deaths in children aged 1 month to 5 years.^29^

RMNCH funding was highly concentrated among a small number of donors, notably the USA, Global Fund, Gavi, the UK, and the Gates Foundation. Because the Global Fund and Gavi channel money from external sources, notably the USA, the UK, and the Gates Foundation, aid for RMNCH is highly vulnerable to political changes in the USA and the UK that can affect foreign aid budgets. Given the important role of the USA's HIV/AIDS funding in improving RMNCH, evidence that this funding decreased substantially in 2018 is particularly concerning.^30^ Private donors provided a small but growing share of RMNCH funding across the years in which they reported; however, the absence of reports from private donors in earlier years limits assessment of changes in funding levels over time.

Muskoka2 generated considerably higher estimates of aid for RMNCH over a longer period than the OECD's RMNCH policy marker. The policy marker relies on donors to report on an additional variable from 2013 onwards, and many donors do this inconsistently or not at all, leading to missing data.^5^, ^13^ The Muskoka2 estimate of $15·4 billion in aid for RMNCH in 2017 was also substantially higher than the IHME estimate of $12·5 billion (constant 2018 USD).^8^ Whereas Muskoka2 aims to estimate the value of aid benefiting RMNCH, IHME aims to estimate the value of aid directly targeting RMNCH. IHME therefore only includes a share of aid directed towards HIV, malaria, or other diseases in its RMNCH estimates if the project descriptions include RMNCH-specific key terms. IHME excludes all funding for the humanitarian and water and sanitation sectors, and also incorporates additional data sources and imputations in its estimates.^5^, ^31^ The conceptualisation of RMNCH inherent within Muskoka2 mirrors the Every Woman Every Child Global Strategy^4^ and reflects the interconnectedness of many health priorities. Such an approach might encourage donors to deploy their resources to achieve multiple priorities simultaneously, such as focusing funds earmarked for HIV on pregnant women and children at risk, or using funds for strengthening of health systems to improve emergency obstetric referrals and birth registration. A siloed approach, limited to specific activities within a given sector, places RMNCH in competition for funding with diseases, health-system strengthening, and other activities, rather than encouraging identification of the links between them.

Our analysis has several limitations. We do not analyse domestic financing, which is crucially important in addressing the financing gap for RMNCH, especially in growing economies with greater resources. The development of Muskoka2 benefited from an extensive stakeholder consultation process; however, different participants could have led to different methodological choices. For example, aid to the education sector was not included, even though women's education is associated with lower child mortality,^32^, ^33^ because the relationship was considered to be less direct than that of the included sectors. Muskoka2 generates reasonably granular estimates but cannot accurately reflect the contributions to RMNCH of individual projects. The percentages used within Muskoka2 are based on numerous assumptions, and in some cases on analyses of historical data (2003–13).^7^ Although historical data might not reflect current disbursement patterns, they reflect the best available evidence and allow for a timely, transparent, and predictable algorithm. In addition, these historical data informed only RMNCH estimates from Gavi, UNICEF, and UNFPA and for humanitarian aid, which together comprised 18% of our RMNCH estimate in 2017. We assumed that regional and unspecified funds followed country-specific funding; however, the use of these funds is likely to vary by donor and possibly over time in ways that cannot be reflected in a simple algorithm. We assumed that core contributions to multilateral institutions were disbursed from those multilaterals in the same year they were received, whereas time lags could exist between receipt and disbursement. Furthermore, use of age-specific and sex-specific disease burden data could underestimate the share of malaria, HIV/AIDS, and tuberculosis funding that benefits RMNCH. Finally, Muskoka2 only includes data from donors that report to the CRS; although the numbers of countries and private foundations reporting to the CRS have increased dramatically in recent years, some donors, including China and Brazil, do not yet do so, and others only report for the most recent years. The Global Financing Facility, which began disbursing grants in 2017,^34^ has not yet reported to the CRS. Greater investment in supporting public, transparent, and comparable reporting is needed.

In the future, Muskoka2 should link to other tracking efforts to understand the overlap and additionality of aid for RMNCH with other areas, such as adolescent health and nutrition. Some funding for adolescent health is included within Muskoka2 estimates of aid for reproductive and maternal health, but it is not separately identified. Existing methods to track aid for adolescent health have not accounted for adolescents' substantially different health needs relative to adults and children, nor the substantial gap between their health needs and use of services, and have excluded sexual and reproductive health.^35^ Muskoka2 includes health sector and some humanitarian aid for nutrition, but it does not take the wider, multisectoral perspective of the World Health Assembly nutrition targets,^36^ nor does it restrict its analysis to funding for interventions with proven effectiveness. Further work to compare Muskoka2 disbursement estimates with WHO's estimates of external expenditure on children younger than 5 years and on specific health areas would also be valuable.^37^ The Muskoka2 method could also be applied to CRS commitments data and to flow types that we have excluded from this analysis.

In addition to using Muskoka2 to track their RMNCH financing, donors should seek synergies in their support for RMNCH and other development priorities. Low-income and middle-income countries need to further strengthen efforts to mobilise domestic resources to meet their RMNCH needs, but aid for RMNCH will still be needed, especially for the poorest countries. Donors must therefore continue to be held accountable for their commitments, which include ensuring that sexual, reproductive, maternal, newborn, child, and adolescent health funding remains central to universal health coverage.

## Data sharing

Data and analyses from this study are available indefinitely at https://doi.org/10.17037/DATA.00001526.
