# Supplementary material for: Estimates of aid for reproductive, maternal, newborn, and child health: findings from application of the Muskoka2 method, 2002–17
Source: Lancet Glob Health. 2020 Feb 5;8(3):e374–86. doi: 10.1016/S2214-109X(20)30005-X (PMC7031705; doi:10.1016/S2214-109X(20)30005-X)

THE LANCET
Global Health

Supplementary appendix

This appendix formed part of the original submission and has been peer reviewed. We post it as supplied by the authors.

Supplement to: Dingle A, Schäferhoff M, Borghi J, et al. Estimates of aid for reproductive, maternal, newborn, and child health: findings from application of the Muskoka2 method, 2002–17. *Lancet Glob Health* 2020; published online Feb 5. http://dx.doi.org/10.1016/S2214-109X(20)30005-X.

Contents

I. Additional information on methods **2**

I.1. The development process for Muskoka2 2

I.2. How Muskoka2 innovates beyond Muskoka1: Eight innovations 3

I.3. Generating year-and country-specific imputed percentages for malaria, tuberculosis and HIV/AIDS in Muskoka2 6

I.4. An alternative approach for generating imputed percentages for humanitarian sector purpose codes in Muskoka2 6

II. Additional results **7**

II.1. Estimates of aid for RMNCH based on Muskoka2 7

II.3. Comparison of M2 estimates with M1A and M1B 22

List of figures

Supplementary figure 1 Aid for RMNCH in the context of all-sector aid and aid for relevant sectors 13

Supplementary figure 2 Aid for RMNCH and aid for other relevant sectors and purposes, 2002-2017 14

Supplementary figure 3 Proportions of aid for RMNCH disbursed for reproductive health, maternal and newborn health, and child health, 2002-17 15

Supplementary figure 4 Aid for RMNCH by recipient country income group, 2002-2017 16

Supplementary figure 5 Aid for RMNCH by purpose code, 2002-17 17

Supplementary figure 6 Aid for reproductive health, maternal and newborn health, and child health, by purpose code, 2002-17 18

Supplementary figure 7 Aid for RMNCH by donor, 2002-17 19

Supplementary figure 8 Aid for RMNCH by income group, 2002-17 20

Supplementary figure 9 Comparison of Muskoka2 and Muskoka1 global estimates of aid for RMNCH, 2002-2017 22

Supplementary figure 10 Comparison of the value of aid for 3 diseases and general budget support counted towards Muskoka2 and Muskoka1 estimates of aid for RMNCH, 2002-2017 ... 23

Supplementary figure 11 Comparison of Muskoka2 and Muskoka1 estimates of aid for RMNCH from 20 largest donors, 2002-2017 24

Supplementary figure 12 Comparison of Muskoka2 and Muskoka1 estimates of aid for RMNCH for 16 recipient countries with greatest RMNCH needs, 2002-2017 25

List of tables

Supplementary table 1 Donors reporting in 2002 and 2017 7

Supplementary table 2 Donors reporting in 2012 and 2017 8

Supplementary table 3 Donors reporting in 2016 and 2017 10

Supplementary table 4 Donors reporting RMNCH disbursements in 2017 but not 2016 12

Supplementary table 5 The 20 countries receiving the most aid for RMNCH in 2017 21

I. Additional information on methods

I.1. The development process for Muskoka2

The Technical Working Group for Tracking Finance for Sexual, Reproductive, Maternal, Newborn, Child and Adolescent Health (SRMNCAH) was established in May 2018 by PMNCH and Countdown to 2030, following a meeting of experts, donors and stakeholders on strengthening tracking of aid and domestic finance for RMNCH. The remit of the Working Group includes to support the development of a single, improved method for tracking aid for RMNCH, and to work with donors to promote the use of the new method. The Working Group met twice, in June and October 2018, to this end.

An initial version of the Muskoka2 method was presented to the Working Group in June 2018. Key elements of the method were discussed with the group, and four decisions were made regarding Muskoka2: 1) the method should include aid disbursements from private foundations, 2) the method should include aid disbursements to the humanitarian sector, using fixed imputed percentages for each of the five humanitarian sector purpose codes, 3) aid disbursements from three multilateral institutions (GAVI, UNFPA and UNICEF) with RMNCH-specific mandates should be treated differently to other donors, with percentages applied to *all* disbursements from these donors, 4) LSHTM should test using year- and country-specific imputed percentages for malaria, HIV/AIDS, tuberculosis and general budget support purpose codes, rather than fixed percentages for these purpose codes as per Muskoka1.

The Working Group met again in October 2018, at which a more refined version of Muskoka2 was presented to the group, incorporating the decisions made at the June meeting, along with preliminary results from applying Muskoka2 to CRS aid data for 2002-2016, and comparing these to results applying Muskoka1 for the same period. At the October meeting it was decided that year- and country-specific imputed percentages for malaria, HIV/AIDS and tuberculosis purpose codes should draw on data on disease morbidity (number of cases), rather than mortality, or funding for disease prevention (see Web Appendix). There was also discussion regarding approaches for allocating regional and unspecified funding to aid estimates for individual recipient countries. It was proposed for the latter that Muskoka2 adopts the Countdown to 2015 approach, whereby countries are assumed to receive a share of regional funding in the same proportion as their country-specific funding within the region that year. For example, Nigeria received 11% of all country-specific funding amongst countries within Sub-Saharan Africa in 2017, so we assumed that also received 11% of regional funding to Sub-Saharan Africa in 2017.

The October 2018 meeting of the Working Group resulted in finalisation of the Muskoka2 method, with agreement on all eight innovations within the method. In November, the Working Group presented recommendations to improve the tracking of aid for SRMNCAH to

the OECD's Working Party on Development Finance Statistics, including use of the Muskoka2 method. Estimates of aid for RMNCH using Muskoka2 for 2002-2016 were presented at the at the PMNCH Partner's Forum in New Delhi in December 2018.

I.2. How Muskoka2 innovates beyond Muskoka1: Eight innovations

The Working Group agreed to eight key methodological changes to Muskoka1 to produce Muskoka2:

1. **Recipient- and year-specific imputed percentages:** In Muskoka1, fixed imputed percentages were applied to all CRS purpose codes; for example, 88.5% of the value of aid with the malaria control purpose code were counted towards RMNCH estimates. This approach did not account for the large variations in how much funding for each of these four areas benefits RMNCH across recipient countries and years. For example, in some countries, the malaria burden is borne almost exclusively by children and pregnant women, while in others, it has shifted to an older age range. Further, some countries allocate a much greater share of general government expenditure to health than other countries. To account for this variation, in Muskoka2, the imputed percentages for four purpose codes - malaria, HIV/AIDS, tuberculosis, and general budget support - vary based on data on the disease burden,[16,17] demography,[16,17] and government health expenditure[18] in each recipient country and year (Figure 1). The selection of these four purpose codes – and not others – is consistent with the previous Countdown approach[6] and reflects the specific nature of these four health areas, the large role of this funding in promoting RMNCH, and data availability.

2. **Disaggregation of estimates by beneficiary group:** Muskoka1 produced estimates of aid for RMNCH in total, but not separately for reproductive health (RH), maternal and newborn health (MNH), and child health (CH). Disaggregating aid for RMNCH is important for alignment with SDG targets, for tracking equity between groups, and for estimating aid per person. For Muskoka2, we split all RMNCH imputed percentages into three separate imputed percentages for RH (defined as reproductive health of non-pregnant women), MNH (defined as health of pregnant and postpartum women and babies under one month old), and CH (defined as health of children aged one month to five years) to allow us to estimate aid for these beneficiary groups separately. (Figure 1) For malaria, HIV, tuberculosis, and general budget support funding, our approach already reflected these separate population groups. To disaggregate our fixed RMNCH imputed percentages, we analysed the Countdown to 2015 ODA+ dataset[6] to determine the proportion of aid for RMNCH that the Countdown approach estimated would benefit RH, MNH, and CH within each purpose code and for each of the three institutions with RMNCH-specific mandates (described in point 6 below).

3. **Regional and unspecified recipients:** Within the CRS, donors categorise the recipient of each disbursement as either a specific recipient country, a geographic region, or an unspecified recipient. As shown in the results, more than a quarter of aid for RMNCH went to regional and unspecified recipients. As Muskoka1 was designed from a donor perspective, it did not specify methods to include regional or unspecified funding within estimates of aid for individual recipient countries. In Muskoka2, a share of all funding for regional and unspecified recipients is included within estimates for individual recipient countries. We used the same approach as the Countdown to 2015: countries are assumed to receive regional and unspecified funds in the same proportion as country-specific funding. For example, Nigeria received 11% of all country-specific RMNCH funding for Sub-Saharan African countries in 2017, so we assumed that Nigeria also benefitted from 11% of regional RMNCH funding for Sub-Saharan Africa in 2017.

4. **Humanitarian funding:** Muskoka1 excluded all disbursements to the humanitarian sector from its estimates of aid for RMNCH; however, humanitarian aid often supports health activities, including those benefitting RMNCH, and this omission may substantially affect estimates for crisis-affected recipient countries. We therefore developed imputed percentages based on the proportion of the value of disbursements in each of the five humanitarian purpose codes that were considered to benefit RMNCH in the Countdown to 2015 ODA+ dataset (2003-2013).[6]

5. **Private flows:** As Muskoka1 was designed to track funding from G8 members, it excluded disbursements from private foundations; however, institutions such as the Bill and Melinda Gates Foundation disburse substantial funds to improve RMNCH. The volume of these disbursements and the number of private foundations reporting their disbursements to the CRS are both increasing. Muskoka2 therefore includes all private development finance reported in the CRS.

6. **Treatment of disbursements from multilaterals:** To generate global and recipient-specific estimates of aid for RMNCH, aid from additional bilateral donors (beyond G8 members) and all aid flowing from multilateral institutions' core budgets must also be assessed. In its accountability reports, PMNCH applied Muskoka1's purpose-code-based imputed percentages to disbursements from all bilateral and multilateral donors' aid in the CRS.[12] This approach underestimated RMNCH disbursements from GAVI, UNFPA, and UNICEF because it did not reflect their RMNCH-specific mandates. For Muskoka2, the Working Group therefore agreed that the purpose-code-based imputed percentages should continue to be applied to all bilateral donors' aid and to most multilateral institutions' aid in the CRS. For aid from GAVI, UNFPA, and UNICEF, however, a fixed percentage of aid was considered to benefit RMNCH. These institution-specific imputed percentages reflected the proportion of all disbursements from each of these institutions that was considered to benefit RMNCH in the Countdown ODA+ dataset (Figure 1).

7. **Crediting bilateral donors for core contributions to multilateral institutions:** In estimating aid for RMNCH from G8 member countries, Muskoka1 included both a share of their direct, bilateral aid to recipient countries, and also a share of their core contributions to 10 multilateral institutions. The 10 multilateral institutions estimated these shares themselves as the proportion of their spending in 2009 that benefitted RMNCH. For example, the UK disbursed $408m in 2017 to the Global Fund, which had estimated that 46% of its spending in 2009 benefitted RMNCH; using Muskoka1, the UK would be considered to have contributed $188m in 2017 for RMNCH through its core contributions to the Global Fund. This approach is subject to bias because it is based on multilaterals' internal estimates for a single year a decade ago, excludes contributions to other multilaterals, does not correspond with how Muskoka2 assesses funds flowing out of these multilaterals, and depended on bilateral donors providing their own data on their core contributions.

   For estimates of aid for RMNCH from individual bilateral donors, Muksoka2 therefore captures a share of core contributions to all multilateral institutions included in the OECD's data table, "members' total use of the multilateral system". This table details each bilateral donor's core contributions to each multilateral institution (including the European Commission) from 2011 onwards. Muskoka2 calculates the proportion of core contributions to each multilateral that benefits RMNCH as the proportion of all disbursements from the relevant multilateral that is estimated to benefit RMNCH each year. For example, as 40% of the value of disbursements from the Global Fund in 2017 were considered to support RMNCH, 40% of each bilateral donor's core contributions to the Global Fund in 2017 were counted towards that bilateral donor's RMNCH contribution. The analysis is thus similar to IHME's analysis by "source" of funding.[19] We only apply this approach in comparing individual donors' RMNCH disbursements; estimates of global aid and aid for individual recipient countries are based exclusively on the CRS and therefore avoid double-counting of multilateral aid.

8. **Communication of uncertainty**: Muskoka1 did not reflect any of the uncertainty inherent in estimates of aid for RMNCH. In Muskoka2, we reflect uncertainty in the imputed percentages used by showing global Muskoka2 estimates in the context of aid that entirely supports RMNCH (namely, aid in the "reproductive health", "family planning", "basic nutrition", and "personnel development for population and reproductive health" purpose codes), and all aid for the health, humanitarian aid, and water and sanitation sectors. We suggest that these values serve as useful extreme lower and upper bounds, respectively, on RMNCH estimates. We reflect uncertainty in methods for crediting donors for their core contributions to multilaterals by presenting these estimates separately from donors' direct disbursements to recipients. Similarly, in estimates for individual recipient countries, we distinguish between funding disbursed directly to each country, and regional and unspecified funding that we assume to benefit

each country. Additional sources of uncertainty are described in the discussion, but this approach highlights in a simple way some of the uncertainty in estimates.

I.3. Generating year-and country-specific imputed percentages for malaria, tuberculosis and HIV/AIDS in Muskoka2

Within Muskoka2, we sought to estimate the amount of aid directed towards malaria, tuberculosis, and HIV/AIDS that contributes to RMNCH. At the June 2018 Working Group meeting, it was decided that year- and country-specific data on the number of cases would be used to estimate the proportion of aid directed towards malaria, tuberculosis, and HIV/AIDS to include in Muskoka2 estimates. The proportion of aid for each disease that is considered to contribute to RMNCH reflects the proportion of all cases of each disease that are: a reproductive health problem for non-pregnant women aged 15-49; a maternal or neonatal health problem; or a health problem for children under 5. For HIV/AIDS and tuberculosis, which are chronic conditions, prevalence data is used, while for malaria, case incidence data is used. Muskoka1 used mortality rather than morbidity data for the imputed percentages for each of these diseases and was based on averages across all low- and middle-income countries. We argue that morbidity better reflects where funding is spent, whereas mortality reflects an absence of funding. Using case data may underestimate malaria funding supporting RMNCH, as a large proportion of malaria funding is spent on prevention rather than curative treatment; however, in the absence of sufficient data on funding spent on malaria prevention in all countries and years analysed within Muskoka2 and in recognition of stakeholders' preference for simplicity, we used disease case data for the disease imputed percentages.

I.4. An alternative approach for generating imputed percentages for humanitarian sector purpose codes in Muskoka2

In addition to the approach described in the main paper for including a share of humanitarian funding in Muskoka2 estimates, we also explored a second approach. This alternative approach sought to achieve greater accuracy in identifying relevant humanitarian projects, but was more complex. This alternative approach involved first using a key term search to identify health projects within each of the humanitarian purpose codes, and then applying imputed percentages to estimate the proportion of the value of these humanitarian health projects that could be considered to support RMNCH. However, at the June 2018 Working Group meeting, OECD secretariat members discouraged the use of key term searches as unreliable and donors rejected this approach as overly complex. We therefore adopted the simpler approach described in the main text.

II. Additional results

II.1. Estimates of aid for RMNCH based on Muskoka2

Supplementary table 1 Donors reporting in 2002 and 2017

This table is restricted to those donors that reported non-zero disbursements in both 2002 and 2017. Examining changes over time based on this sub-set of donors addresses the bias that may be introduced when all reported data is considered because some donors have reported their aid in some years but not others and it is not clear if missing data reflects a true absence of disbursements or absence of reporting. Constant 2016 United States dollars (millions) are presented.

Donor	2002	2009	2016	2017
African Development Bank	0	0	0	0
African Development Fund	35	60	18	9
Australia	84	113	93	101
Austria	4	7	8	5
Belgium	47	58	58	59
Canada	59	237	268	337
EU Institutions	71	307	494	528
Finland	12	21	14	17
France	100	129	158	109
Germany	106	225	403	436
Global Environment Facility	0	0	0	0
Greece	2	7	0	1
IMF (Concessional Trust Funds)	51	52	26	30
International Development Association	649	509	634	814
Ireland	39	54	39	45
Italy	8	40	39	63
Japan	71	206	282	308
Korea	0	51	100	100
Luxembourg	1	25	22	19
Netherlands	117	196	279	244
New Zealand	3	13	9	9
Norway	68	112	106	127
Portugal	5	3	6	3
Spain	48	196	30	31
Sweden	67	113	159	177
Switzerland	33	42	72	77
UNAIDS	40	76	64	55
UNFPA	171	162	126	104
UNICEF	114	157	217	225
United Kingdom	261	569	957	1,028
United States	970	3,940	4,616	4,986
**Grand Total**	**3,237**	**7,680**	**9,295**	**10,045**

Supplementary table 2 Donors reporting in 2012 and 2017

This table is restricted to those donors that reported non-zero disbursements in both 2012 and 2017. Examining changes over time based on this sub-set of donors addresses the bias that may be introduced when all reported data is considered because some donors have reported their aid in some years but not others and it is not clear if missing data reflects a true absence of disbursements or absence of reporting. Constant 2016 United States dollars (millions) are presented.

Donors	2012	2016	2017
Adaptation Fund	0	0	0
African Development Bank	0	0	0
African Development Fund	39	18	9
Arab Fund (AFESD)	2	2	1
Asian Development Bank	36	44	40
Australia	213	93	101
Austria	5	8	5
Belgium	59	58	59
Bill & Melinda Gates Foundation	734	1,202	1,281
Canada	326	268	337
Council of Europe Development Bank	2	1	0
Czech Republic	1	2	2
Denmark	60	51	88
EU Institutions	343	494	528
Finland	23	14	17
France	108	158	109
Germany	237	403	436
Global Alliance for Vaccines and Immunization	881	1,218	1,503
Global Environment Facility	0	0	0
Global Fund	1,294	1,490	1,667
Greece	1	0	1
Iceland	1	1	1
IMF (Concessional Trust Funds)	30	26	30
Inter-American Development Bank	11	32	56
International Development Association	399	634	814
Ireland	43	39	45
Islamic Development Bank	0	0	0
Italy	20	39	63
Japan	237	282	308
Korea	63	100	100
Kuwait	4	4	2
Luxembourg	17	22	19
Netherlands	160	279	244
New Zealand	15	9	9
Nordic Development Fund	0	0	0
Norway	87	106	127
OPEC Fund for International Development	15	7	7
Portugal	5	6	3
Slovenia	1	0	0
Spain	40	30	31

Sweden	163	159	177
Switzerland	58	72	77
UN Peacebuilding Fund	1	0	0
UNAIDS	74	64	55
UNDP	11	3	3
UNECE	0	0	0
UNFPA	155	126	104
UNHCR	13	22	23
UNICEF	157	217	225
United Arab Emirates	45	141	142
United Kingdom	931	957	1,028
United States	4,445	4,616	4,986
UNRWA	43	50	61
WFP	15	23	17
World Health Organisation	149	204	199
**Grand Total**	**11,770**	**13,794**	**15,138**

Supplementary table 3 Donors reporting in 2016 and 2017

This table is restricted to those donors that reported non-zero values of aid for RMNCH in both 2016 and 2017. Examining trends over time based on this sub-set of donors addresses the bias that may be introduced when all reported data is considered because some donors have reported their aid in some years but not others and it is not clear if missing data reflects a true absence of disbursements or absence of reporting. Constant 2016 United States dollars (millions) are presented.

Donors	2016	2017
Adaptation Fund	0	0
African Development Bank	0	0
African Development Fund	18	9
Arab Fund (AFESD)	2	1
Asian Development Bank	44	40
Australia	93	101
Austria	8	5
Azerbaijan	0	0
Belgium	58	59
Bill & Melinda Gates Foundation	1,202	1,281
Bulgaria	0	0
Canada	268	337
Caribbean Development Bank	0	0
Council of Europe Development Bank	1	0
Croatia	0	3
Czech Republic	2	2
Denmark	51	88
Dutch Postcode Lottery	11	12
Estonia	0	0
EU Institutions	494	528
Finland	14	17
France	158	109
Germany	403	436
Global Alliance for Vaccines and Immunization	1,218	1,503
Global Environment Facility	0	0
Global Fund	1,490	1,667
Greece	0	1
Hungary	0	0
Iceland	1	1
IFAD	0	0
IMF (Concessional Trust Funds)	26	30
Inter-American Development Bank	32	56
International Development Association	634	814
Ireland	39	45
Islamic Development Bank	0	0
Italy	39	63
Japan	282	308
Kazakhstan	0	0
Korea	100	100
Kuwait	4	2
Latvia	0	0

Lithuania	0	0
Luxembourg	22	19
Malta	0	0
MetLife Foundation	0	0
Netherlands	279	244
New Zealand	9	9
Norway	106	127
OPEC Fund for International Development	7	7
People's Postcode Lottery	1	2
Poland	2	2
Portugal	6	3
Romania	0	0
Saudi Arabia	53	25
Slovak Republic	0	1
Slovenia	0	0
Spain	30	31
Sweden	159	177
Swedish Postcode Lottery	2	2
Switzerland	72	77
Thailand	0	0
Turkey	258	358
UN Peacebuilding Fund	0	0
UNAIDS	64	55
UNDP	3	3
UNECE	0	0
UNFPA	126	104
UNHCR	22	23
UNICEF	217	225
United Arab Emirates	141	142
United Kingdom	957	1,028
United States	4,616	4,986
UNRWA	50	61
WFP	23	17
World Health Organisation	204	199
**Grand Total**	**14,123**	**15,545**

Supplementary table 4 Donors reporting RMNCH disbursements in 2017 but not 2016

Constant 2016 United States dollars (millions) are presented.

**Donor**	**2017**
Bernard van Leer Foundation	3
C&A Foundation	0
Carnegie Corporation of New York	0
Central Emergency Response Fund	15
Charity Projects Ltd (Comic Relief)	11
Children's Investment Fund Foundation	130
Conrad N. Hilton Foundation	3
David & Lucile Packard Foundation	21
Development Bank of Latin America	0
Food and Agriculture Organisation	0
Ford Foundation	4
H&M Foundation	1
John D. & Catherine T. MacArthur Foundation	11
MasterCard Foundation	1
Michael & Susan Dell Foundation	0
Oak Foundation	3
Wellcome Trust	96
William & Flora Hewlett Foundation	34
**Grand Total**	**335**

Supplementary figure 1 Aid for RMNCH in the context of all-sector aid and aid for relevant sectors
This figure presents all-sector aid. The Muskoka2 RMNCH estimates are shown as a percentage of all-sector aid (dark blue line) and as a percentage of aid for "key sectors" (dark red line). These "key sectors" are the health and population, water and sanitation, and humanitarian sectors combined. RMNCH: Reproductive, maternal, newborn, and child health. USD: United States Dollars.

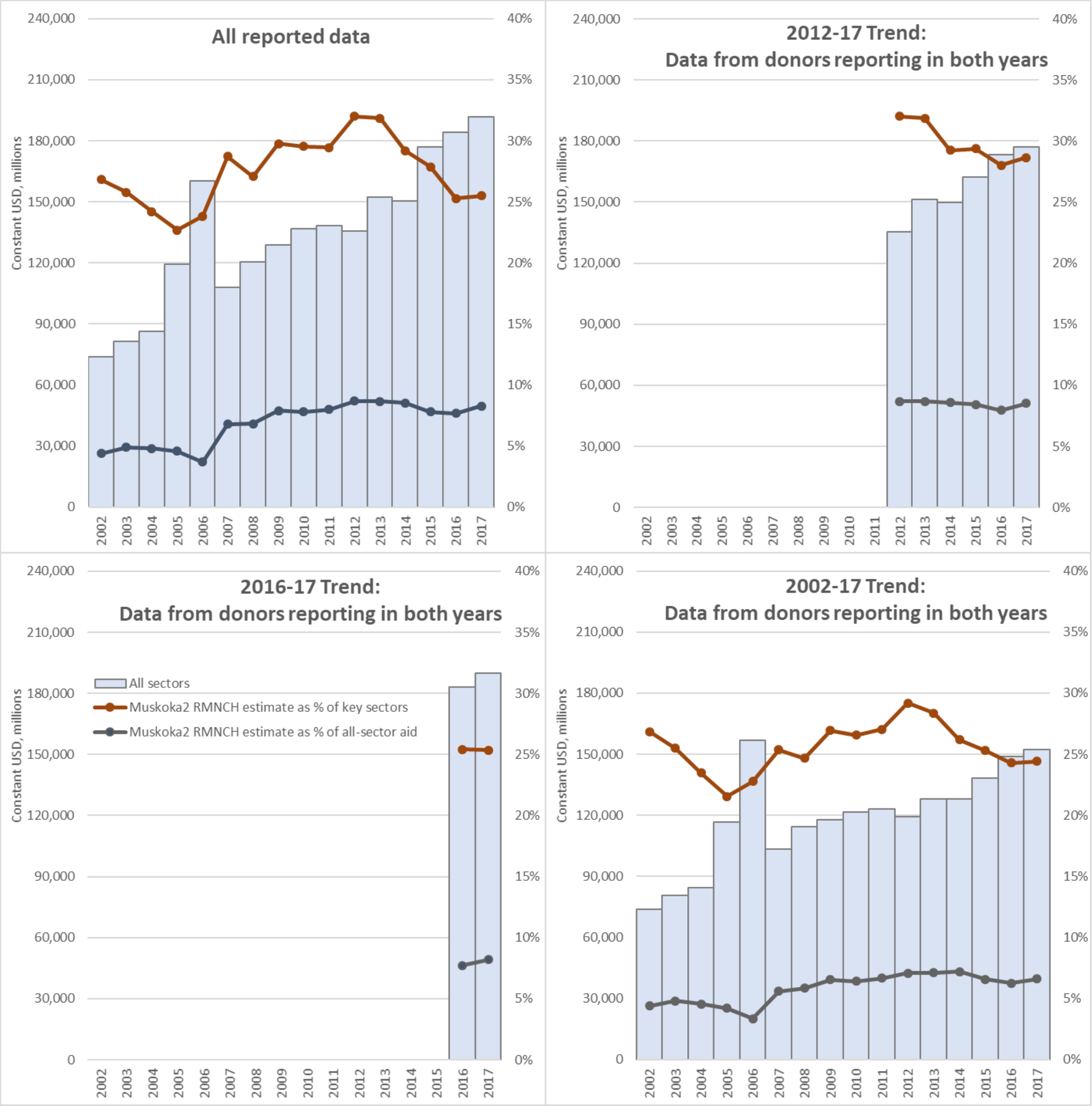

Supplementary figure 2 Aid for RMNCH and aid for other relevant sectors and purposes, 2002-2017

This figure presents the Muskoka2 RMNCH estimates in the context of aid categorized according to relevant purpose codes and sectors in the OECD's CRS dataset. The four "RMNCH-specific purpose codes are: "family planning", "reproductive health", "basic nutrition", and "personnel development for population policy and reproductive health"; 100% of the value of funding in these purpose codes is included in Muskoka2 estimates of aid for RMNCH. In addition, aid categorized within the OECD's definition of the health and population sectors, water and sanitation sector, and humanitarian sector are shown separately and combined. RMNCH: Reproductive, maternal, newborn, and child health. USD: United States Dollars.

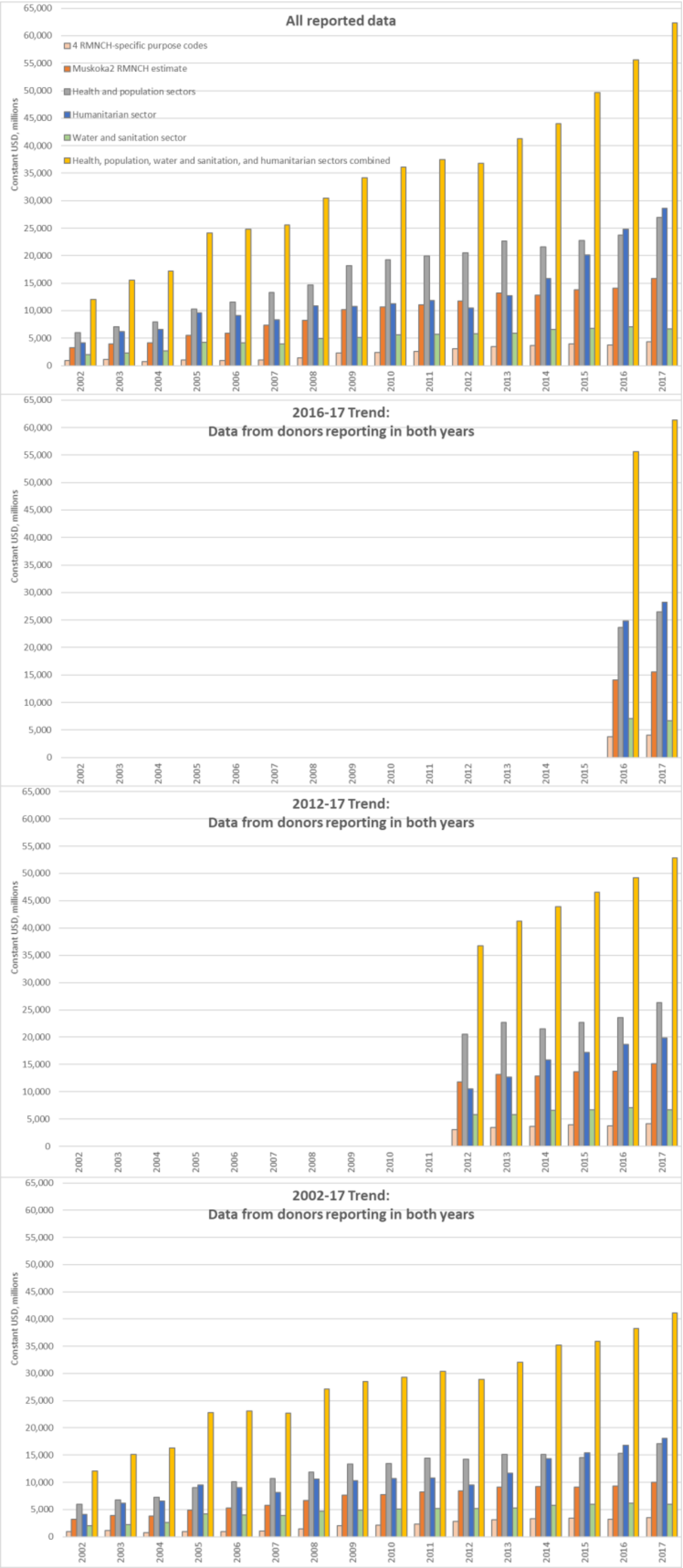

Supplementary figure 3 Proportions of aid for RMNCH disbursed for reproductive health, maternal and newborn health, and child health, 2002-17

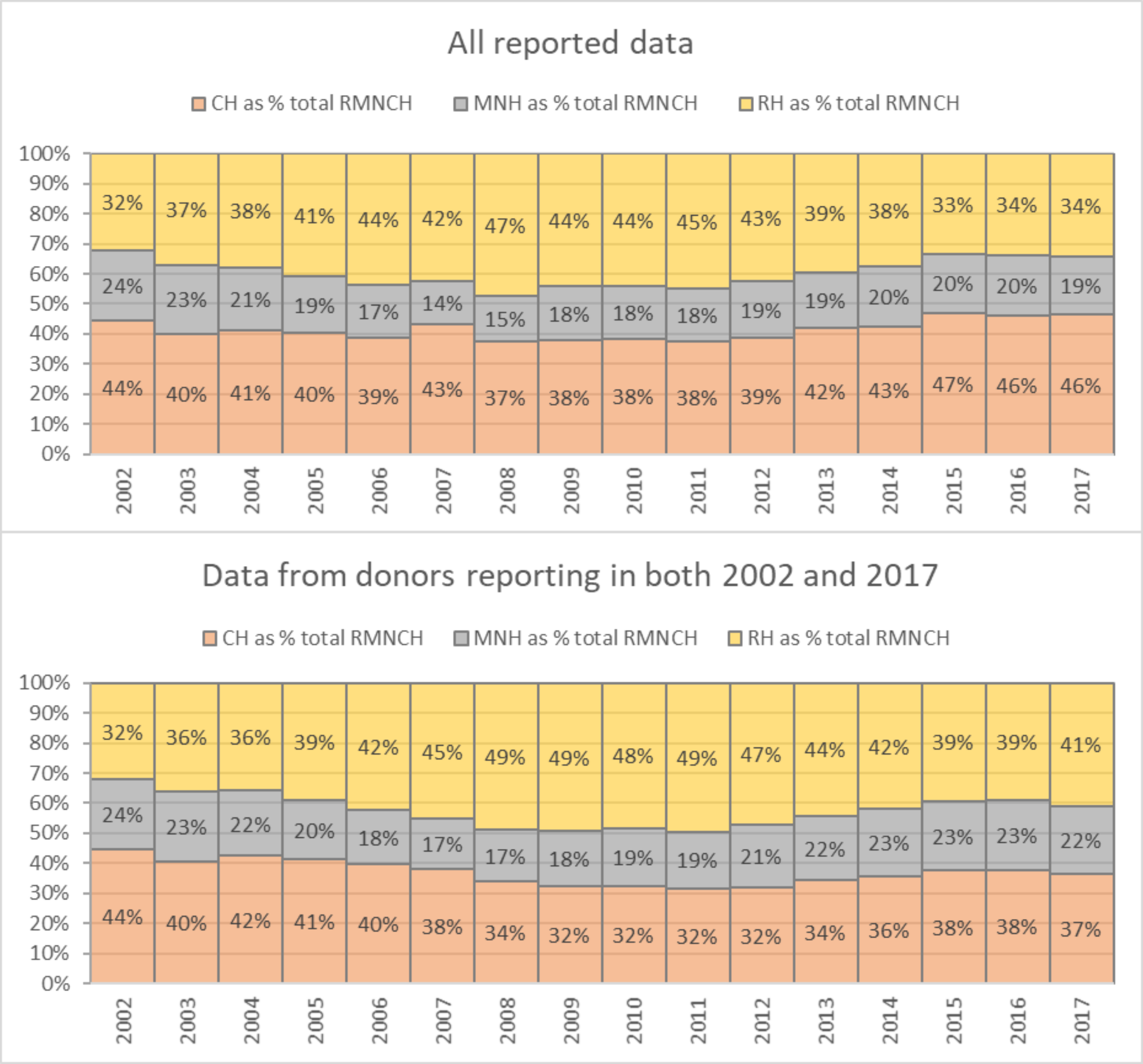

Supplementary figure 4 Aid for RMNCH by recipient country income group, 2002-2017

Data are restricted to donors that reported in both 2002 and 2017.

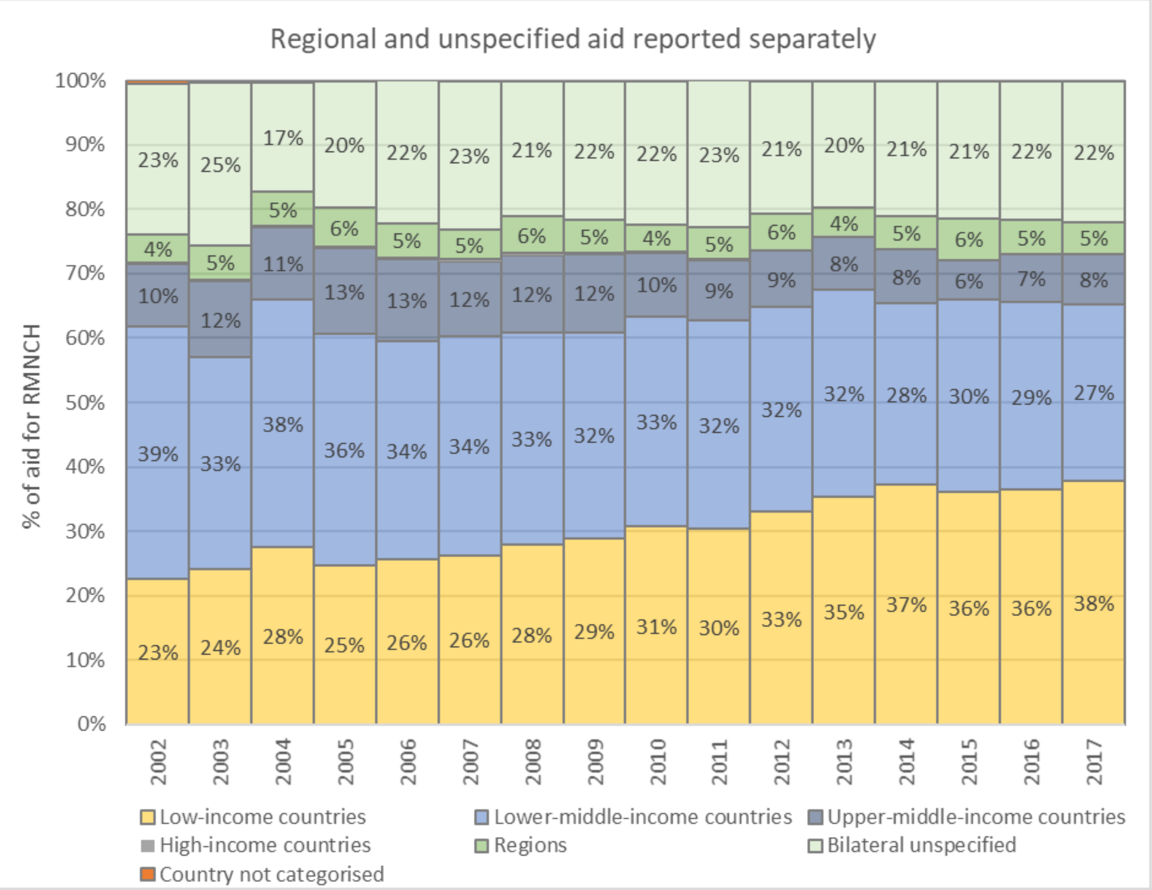

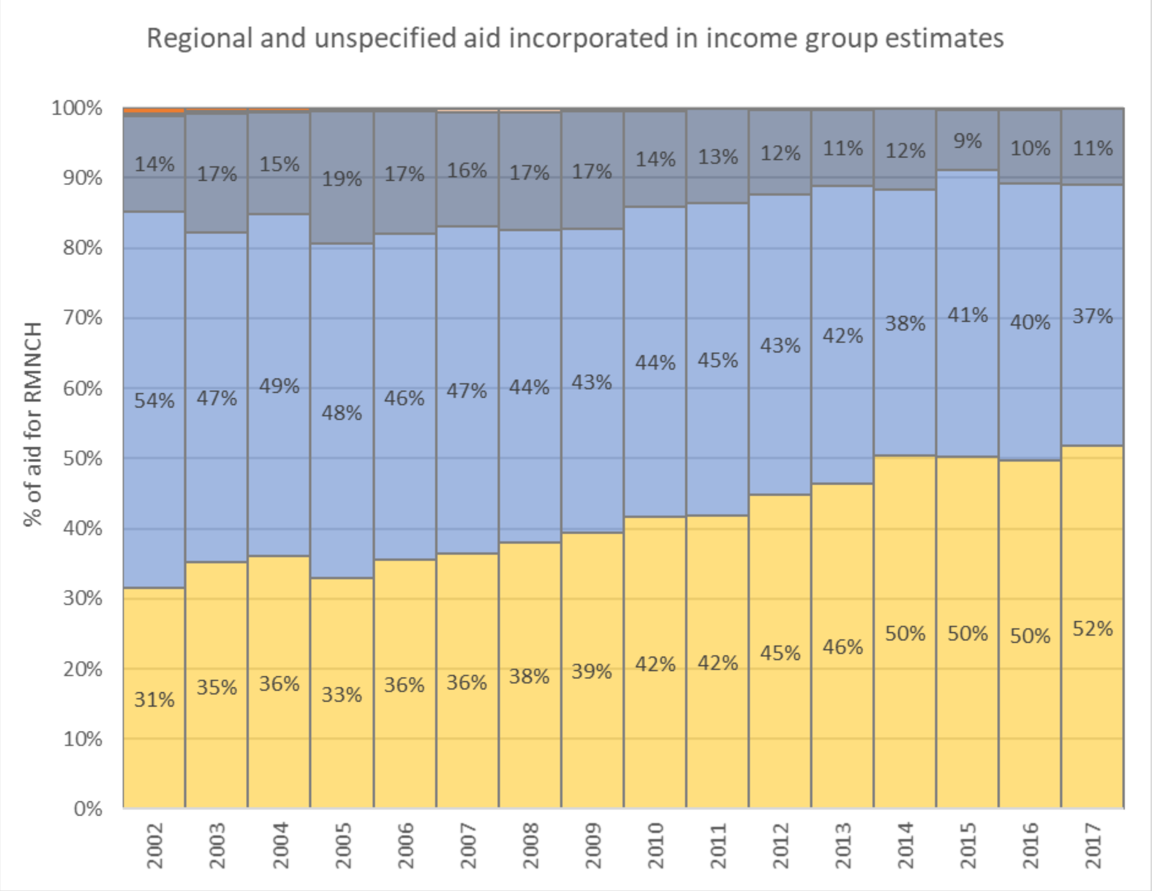

Supplementary figure 5 Aid for RMNCH by purpose code, 2002-17

Largest purpose codes labelled.

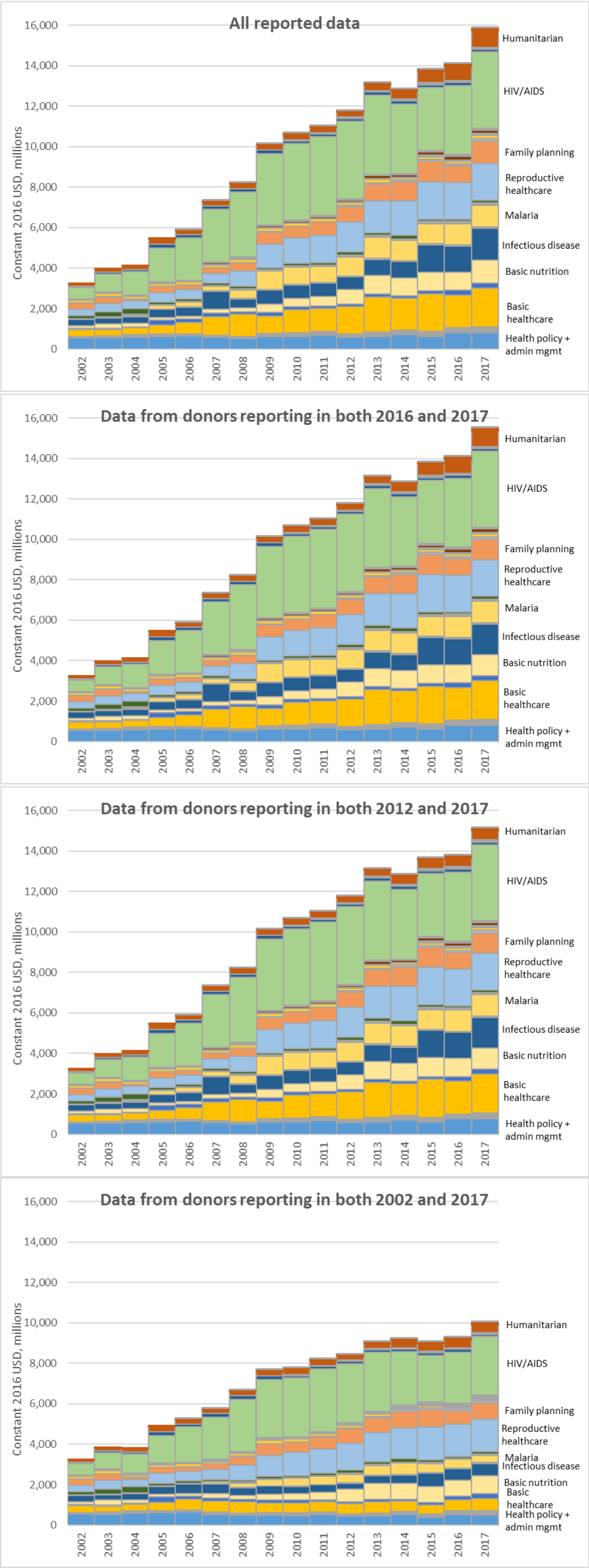

Supplementary figure 6 Aid for reproductive health, maternal and newborn health, and child health, by purpose code, 2002-17

All reported data. Largest purpose codes labelled.

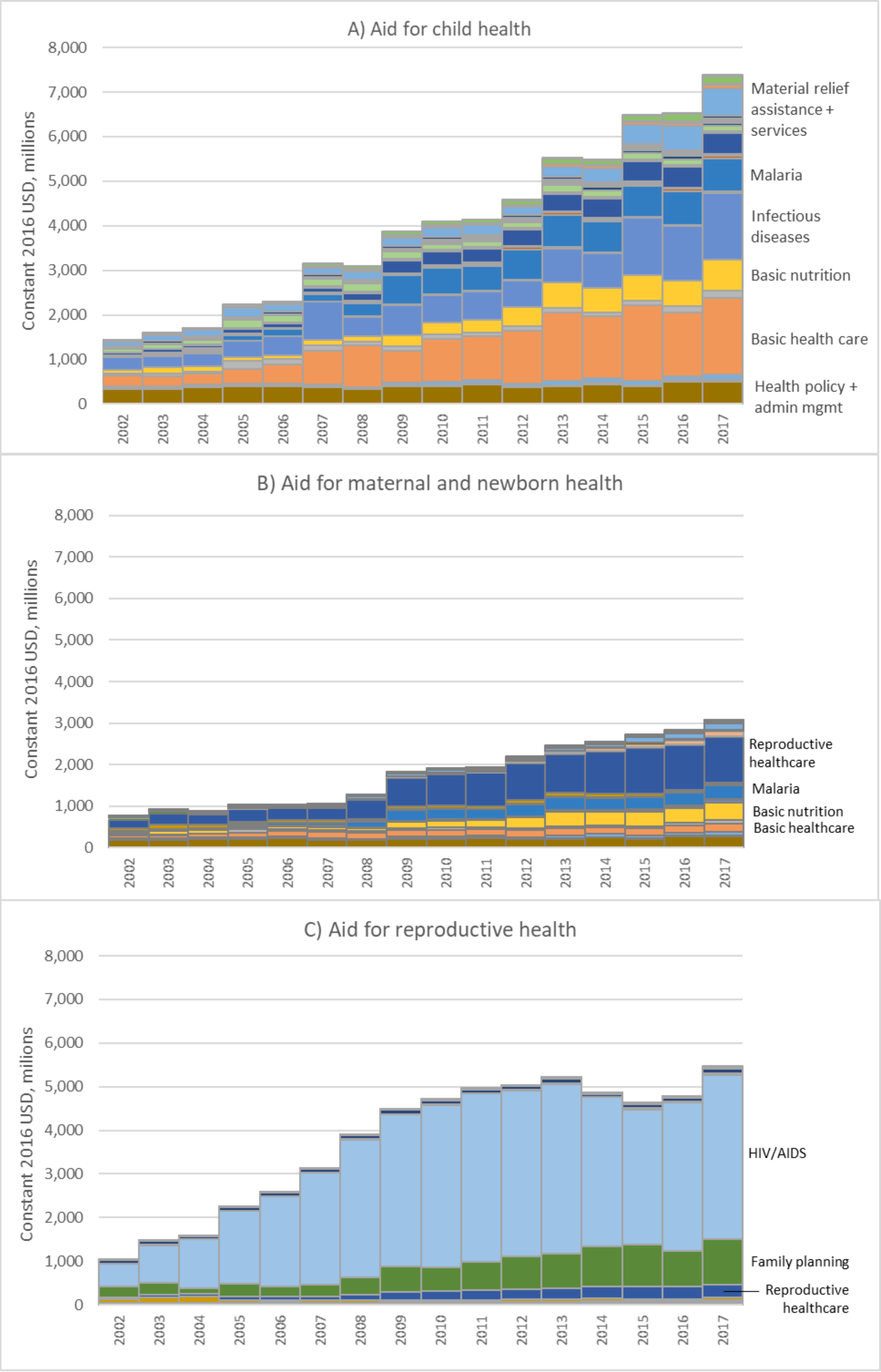

Supplementary figure 7 Aid for RMNCH by donor, 2002-17
All reported data. The eight largest donors are labelled. GAVI: Global Alliance for Vaccines and Immunization.

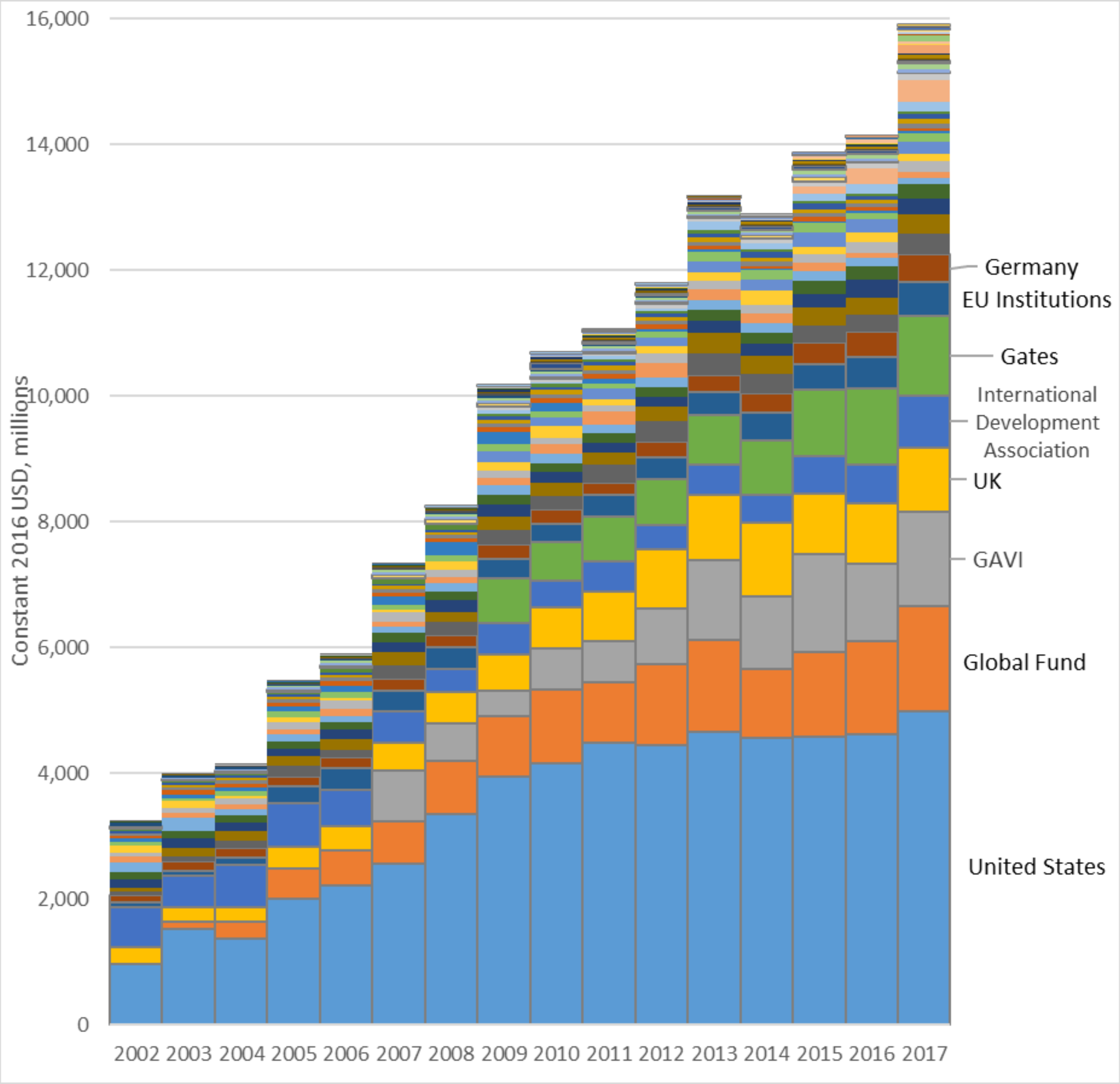

Supplementary figure 8 Aid for RMNCH by income group, 2002-17

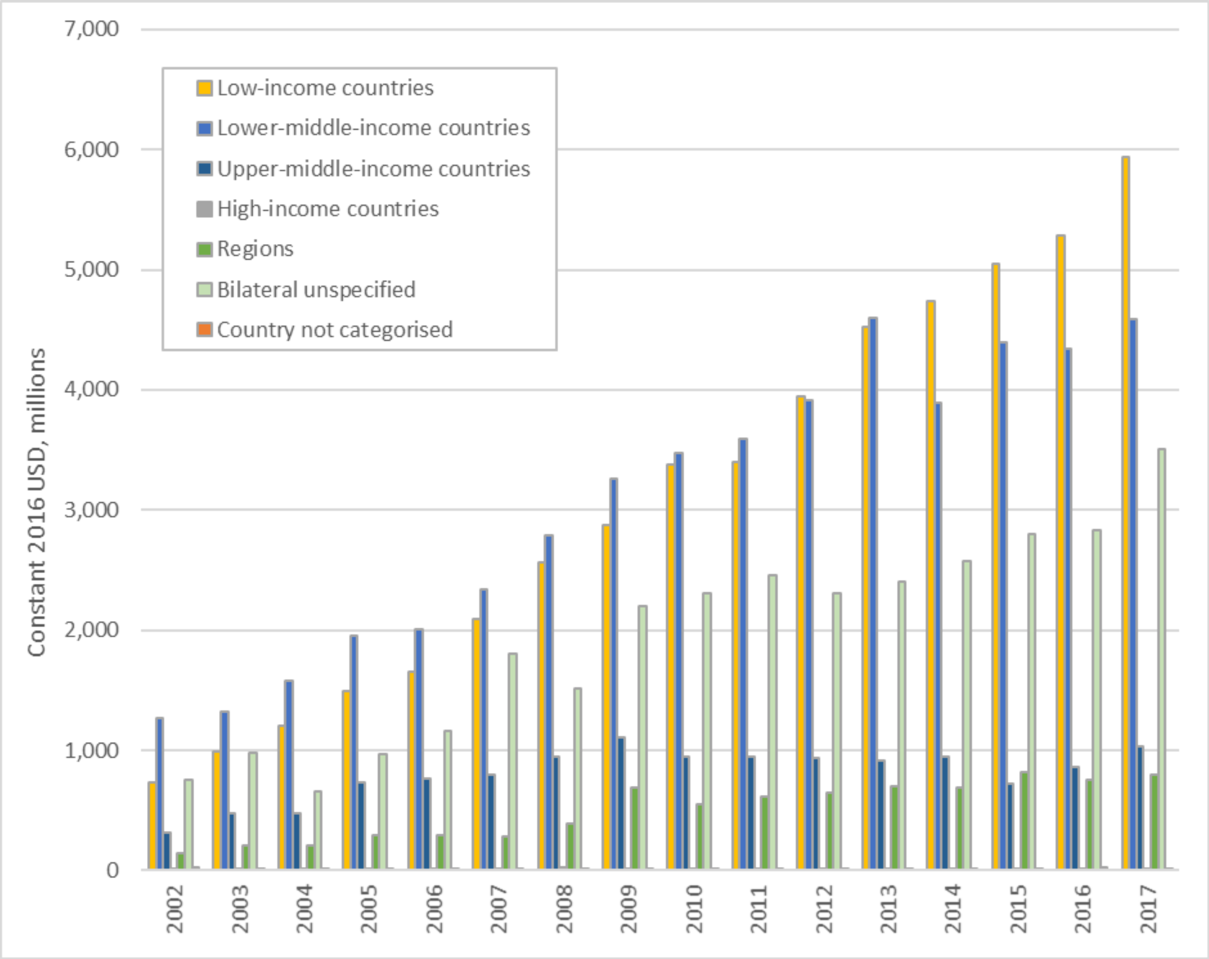

Supplementary table 5 The 20 countries receiving the most aid for RMNCH in 2017
Constant 2016 United States dollars (millions) presented.

Rank	Country	Country-specific aid for reproductive health	Country-specific aid for maternal and newborn health	Country-specific aid for child health	Share of regional and unspecified aid	Total aid for RMNCH	% of global aid for RMNCH
1	Nigeria	$274	$143	$385	$303	$1,107	7%
2	Ethiopia	$230	$149	$256	$240	$876	6%
3	Kenya	$344	$70	$133	$207	$754	5%
4	Tanzania	$284	$91	$171	$206	$751	5%
5	Democratic Republic of the Congo	$119	$87	$247	$171	$623	4%
6	Syrian Arab Republic	$10	$95	$338	$156	$599	4%
7	India	$91	$85	$263	$144	$583	4%
8	Mozambique	$236	$59	$134	$162	$590	4%
9	Uganda	$238	$51	$126	$157	$573	4%
10	Pakistan	$64	$63	$247	$123	$496	3%
11	Zambia	$172	$39	$104	$119	$434	3%
12	South Africa	$263	$5	$41	$117	$426	3%
13	Yemen	$20	$100	$181	$106	$407	3%
14	Bangladesh	$60	$68	$167	$97	$391	2%
15	Afghanistan	$45	$102	$138	$93	$378	2%
16	Malawi	$86	$57	$141	$107	$392	2%
17	South Sudan	$32	$68	$125	$85	$310	2%
18	Zimbabwe	$128	$28	$56	$80	$292	2%
19	Indonesia	$40	$35	$97	$62	$233	1%
20	Mali	$39	$43	$84	$63	$230	1%

II.3. Comparison of M2 estimates with M1A and M1B

We compared estimates of aid for RMNCH using Muskoka2 and Muskoka1 to understand the impact of the eight innovations that we have brought into Muskoka2 on aid estimates. The original Muskoka1 methodology was intended for use by the G8 countries only.

Supplementary figure 9 Comparison of Muskoka2 and Muskoka1 global estimates of aid for RMNCH, 2002-2017

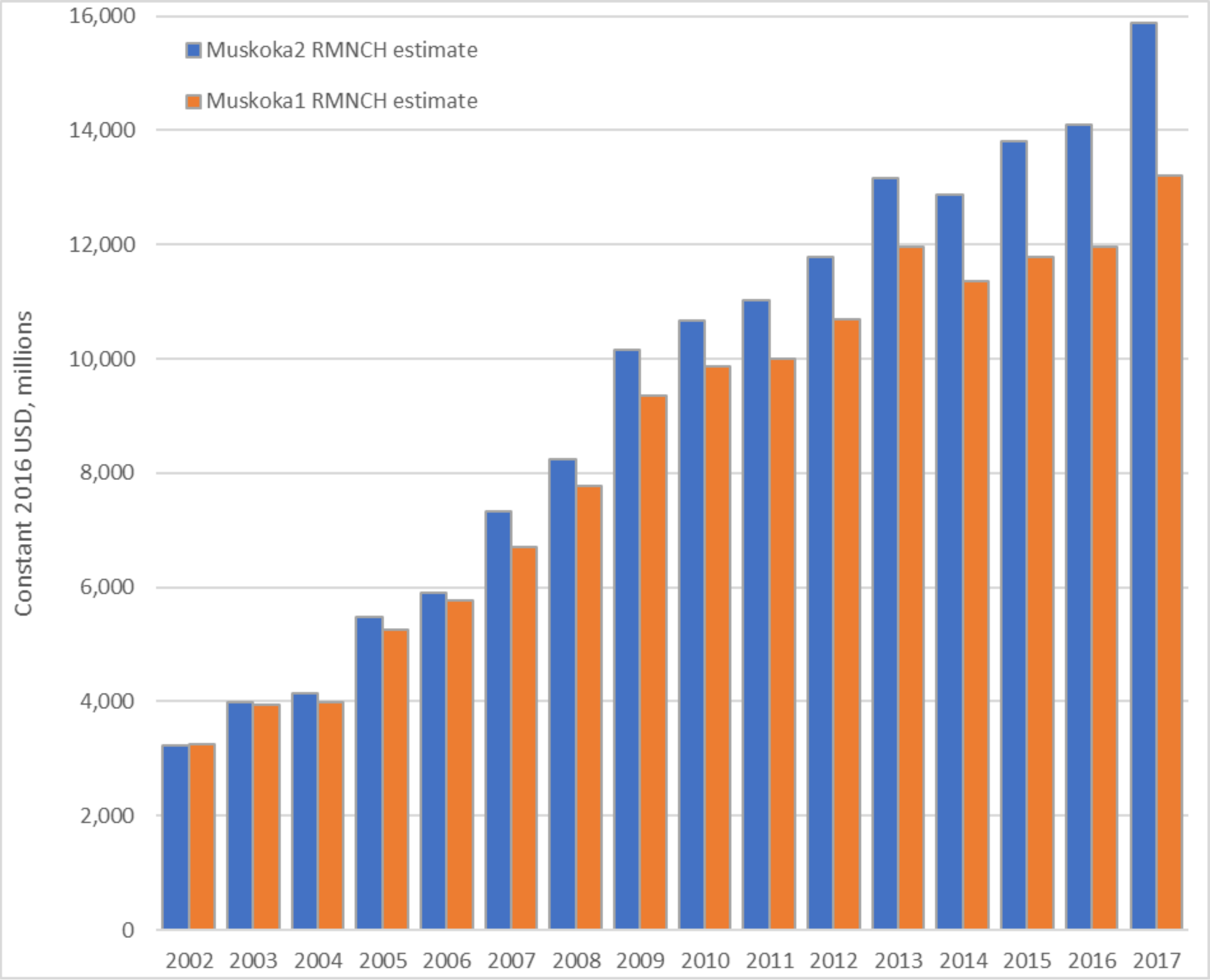

Supplementary figure 10 Comparison of the value of aid for 3 diseases and general budget support counted towards Muskoka2 and Muskoka1 estimates of aid for RMNCH, 2002-2017

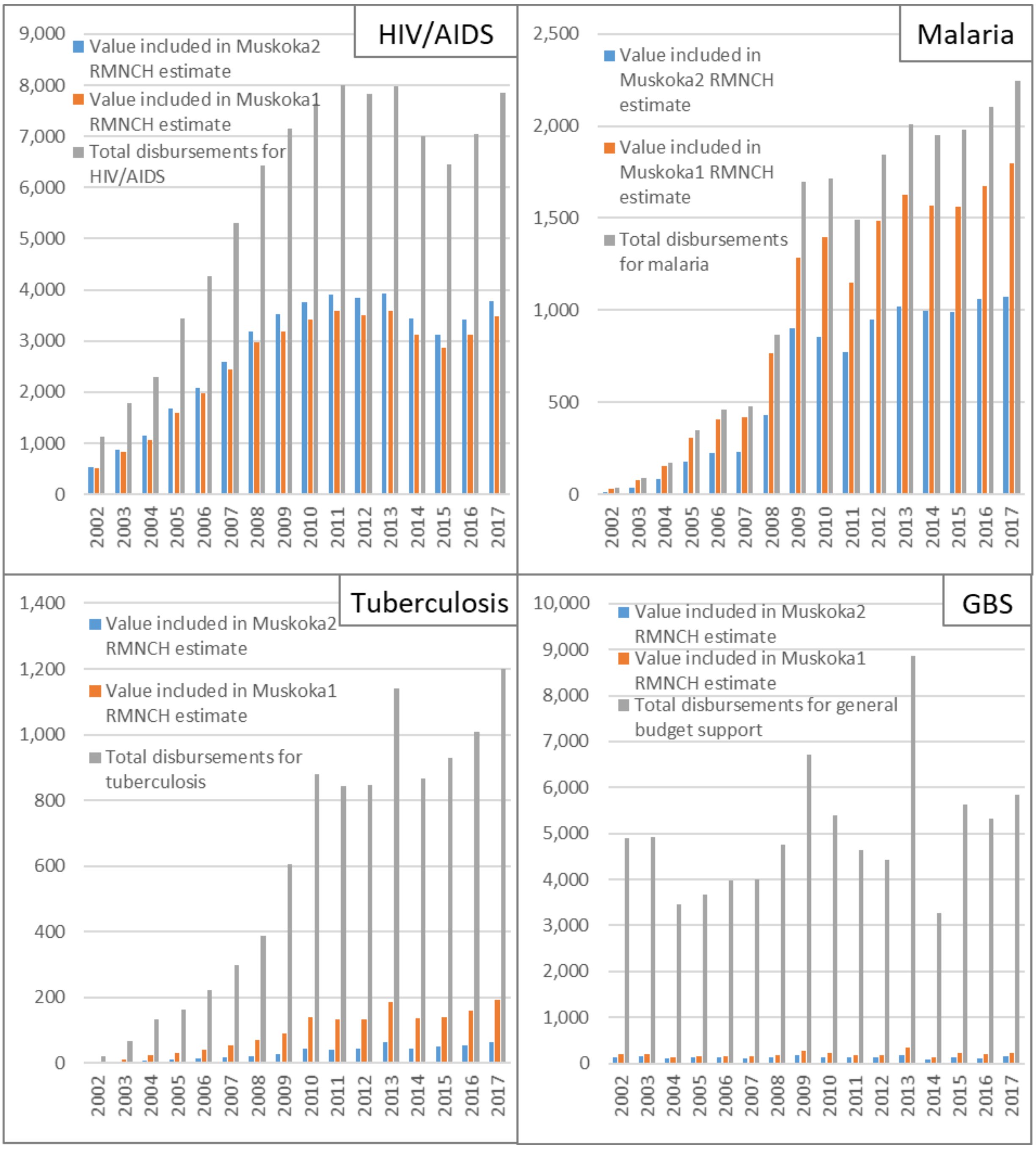

Supplementary figure 11 Comparison of Muskoka2 and Muskoka1 estimates of aid for RMNCH from 20 largest donors, 2002-2017

Shares of core contributions to multilaterals are not included in this comparison, as Muskoka1 relied on data not publicly available through the OECD's International Development Statistics database. Muskoka2 estimates incorporating relevant shares of core contributions to multilaterals within bilateral donors' estimates are provided in the main paper.

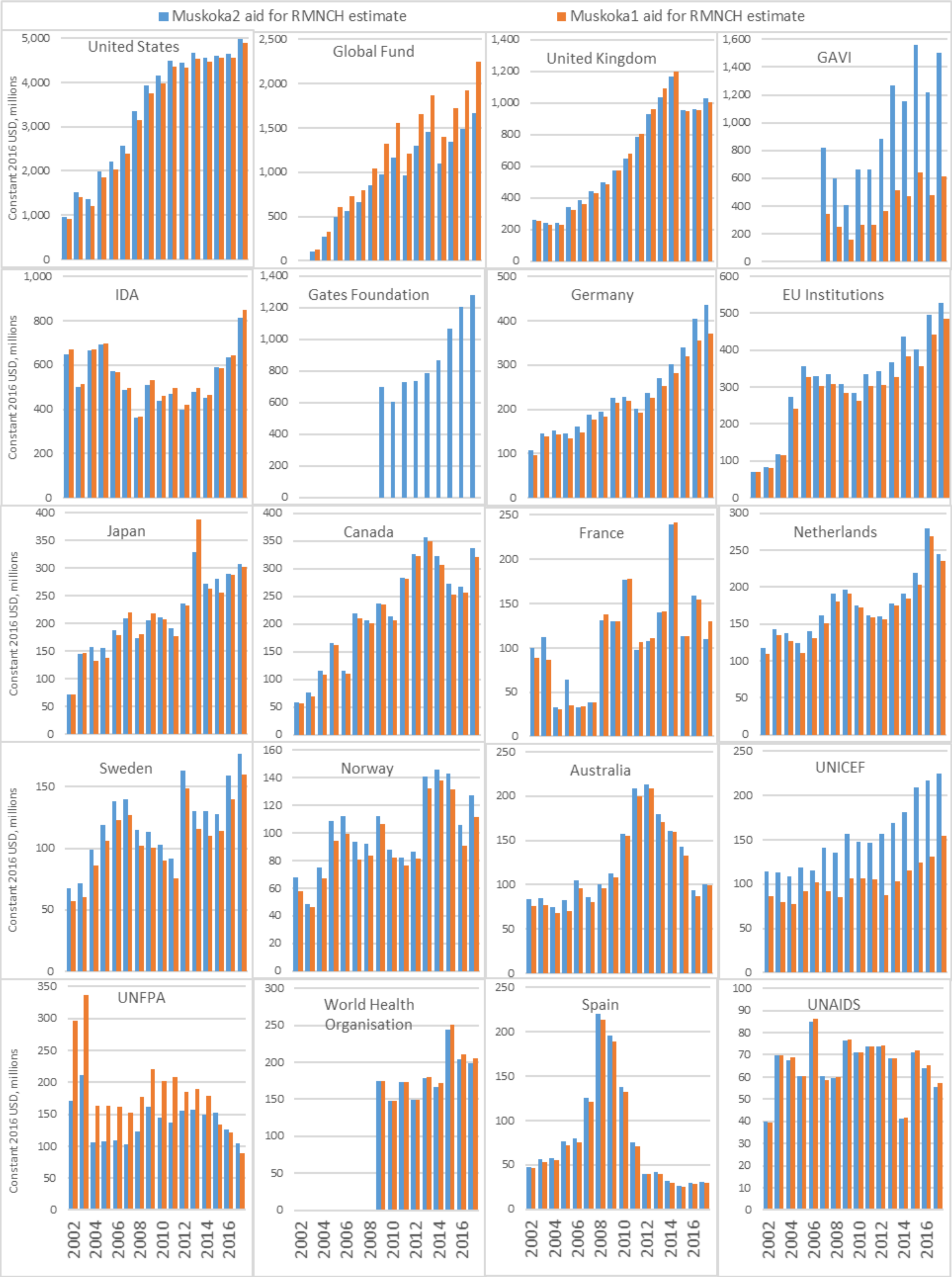

Supplementary figure 12 Comparison of Muskoka2 and Muskoka1 estimates of aid for RMNCH for 16 recipient countries with greatest RMNCH needs, 2002-2017

The Muskoka2 estimates presented here are based only on disbursements specifically for the named recipient country; relevant shares of funding for regional and unspecified recipients are not included and would further increase the Muskoka2 estimates for all recipients, as shown in the main paper. Definition of "RMNCH need" is provided in methods section of main paper.

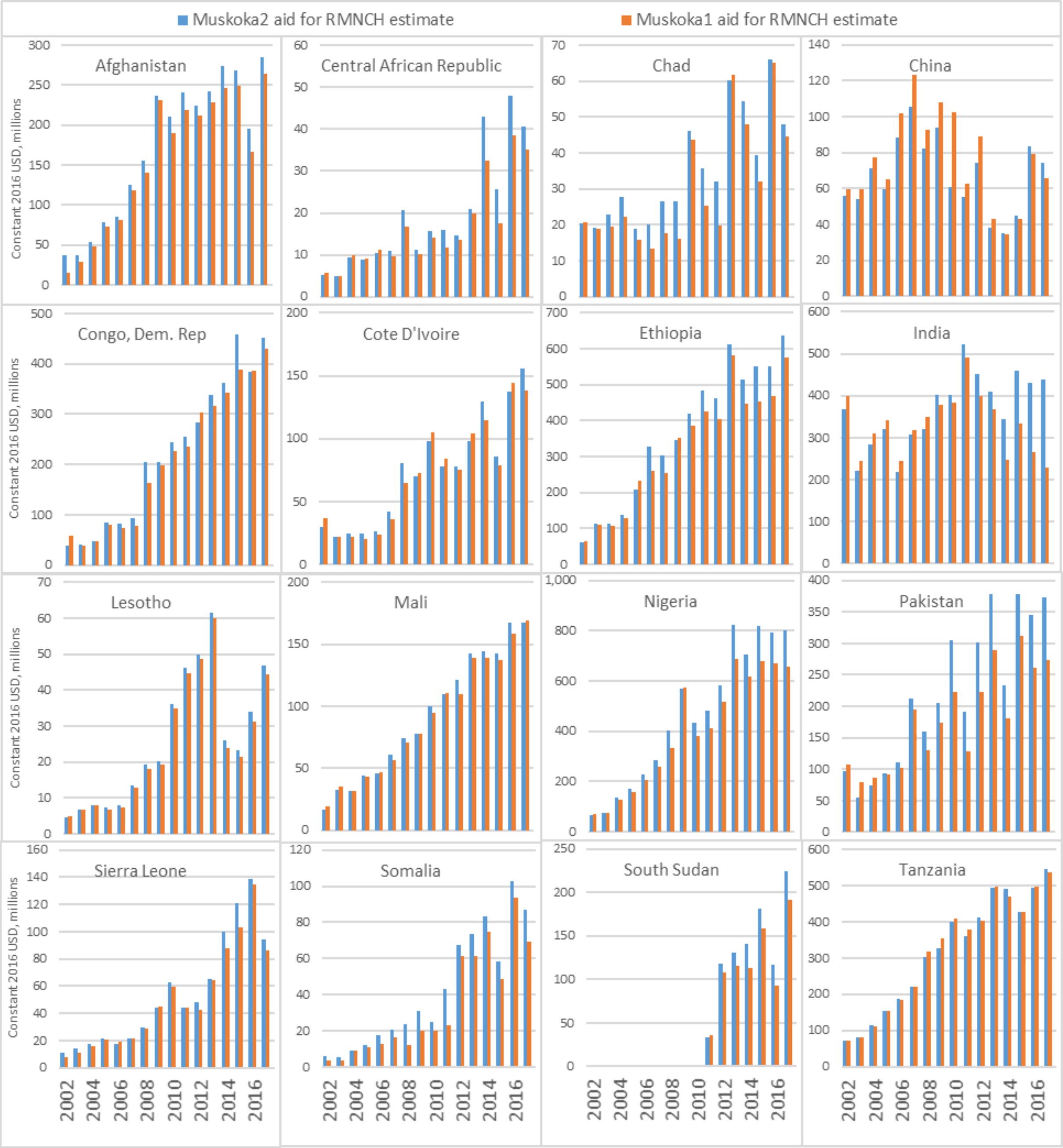

Supplement: Supplementary appendix [file mmc1.pdf]
